# Breast cancer is marked by specific, Public T-cell receptor CDR3 regions shared by mice and humans

**DOI:** 10.1371/journal.pcbi.1008486

**Published:** 2021-01-19

**Authors:** Miri Gordin, Hagit Philip, Alona Zilberberg, Moriah Gidoni, Raanan Margalit, Christopher Clouser, Kristofor Adams, Francois Vigneault, Irun R. Cohen, Gur Yaari, Sol Efroni

**Affiliations:** 1 The Mina & Everard Goodman Faculty of Life Sciences, Bar Ilan University, Ramat-Gan, Israel; 2 Faculty of Engineering, Bar Ilan University, Ramat Gan, Israel; 3 Science in Action Ltd., Ness Ziona, Israel; 4 Juno Therapeutics, Seattle, Washington, United States of America; 5 Department of Immunology, The Weizmann Institute of Science, Rehovot, Israel; University College London, UNITED KINGDOM

## Abstract

The partial success of tumor immunotherapy induced by checkpoint blockade, which is not antigen-specific, suggests that the immune system of some patients contain antigen receptors able to specifically identify tumor cells. Here we focused on T-cell receptor (TCR) repertoires associated with spontaneous breast cancer. We studied the alpha and beta chain CDR3 domains of TCR repertoires of CD4 T cells using deep sequencing of cell populations in mice and applied the results to published TCR sequence data obtained from human patients. We screened peripheral blood T cells obtained monthly from individual mice spontaneously developing breast tumors by 5 months. We then looked at identical TCR sequences in published human studies; we used TCGA data from tumors and healthy tissues of 1,256 breast cancer resections and from 4 focused studies including sequences from tumors, lymph nodes, blood and healthy tissues, and from single cell dataset of 3 breast cancer subjects. We now report that mice spontaneously developing breast cancer manifest shared, Public CDR3 regions in both their alpha and beta and that a significant number of women with early breast cancer manifest identical CDR3 sequences. These findings suggest that the development of breast cancer is associated, across species, with biomarker, exclusive TCR repertoires.

## Author summary

More than 1,100 cancer treatments are currently in clinical testing. Of these, 295 are immune-oncology medicines and vaccines. For Checkpoint Immunotherapy alone, 52,539 patients were enrolled in 2017. Of these treatments, 108 are for breast cancer, the leading cancer diagnosed in women. Although checkpoint immunotherapy has yet to be proven beneficial in most types of breast cancer, current observations of which specific T cell go into the tumor, and how these cells are connected to the response to treatment, have lead to a new understating of the role of the immune system. Yet, and in spite of this intense research into the role of T cells in tumors, very little is known about how clones of T cells behave during tumor formation. The paper we present here studies this clonal behavior over time. We study it during mammary tumor development in mice. We see that a set of T cell clones, which comes from a diversity of different nucleotide sequences, stands out in its behavior compared with other T cell clones. Importantly, we show that these mouse T cell clones have a strong connection to human breast cancer. The specific identification of a subset of T cell clones, together with the behavior of these clones over time, is critical to our understanding of the effect of modifications to the immune system, such as the modifications made by immunotherapy treatments, and to our understanding of how to better control the behavior of the immune system.

## Introduction

The CDR3 region of the TCR binds the peptide epitopes presented by MHC molecules to antigen-specific T cells; the CDR3 region thus directs T-cell antigen specificity [1]. Despite the astronomical numbers of possible CDR3 nucleotide (NT) recombinations [2], more than 10^18 different TCR sequences, healthy mice and humans manifest shared, Public TCR CDR3 amino acid (AA) sequences that seem to be enriched for associations with self-reactivity, allo-reactivity and tumors [3,4]. These Public TCR repertoires seem to emerge as a consequence of selection and are rich in convergent recombination–different (NT) recombinations encode identical (AA) CDR3 sequences. Notably, prevalent CDR3 regions appear across species in mice and humans [3].

The present study was done to test whether the spontaneous development of a tissue-specific tumor might be associated with detectible Public TCR CDR3 sequences; such tumor-associated sequences could function in T-cells that might mediate tumor immunity or, alternatively, might protect tumors by immune suppression [5]. The first question, however, was whether a developing tumor, in the absence of premeditated immunization, might induce or enhance the expression of tumor-associated CDR3 TCR sequences. The use of multiple sequences across individual animals, different phenotypes groups and across species, led us to use the term “Public” in the context of this manuscript, to indicate CDR3 sequences that appear in two animals, or in two subjects. Since mice and humans have been found to share prevalent Public TCR sequences, we carried out this study in two stages: In the first stage we carried out a longitudinal study of TCR repertoires in mice undergoing the development of spontaneous, transgene-engineered breast cancer; the results uncovered multiple CDR3 regions sequences that were Public and Exclusive to mice developing tumors; these sequences were undetectable in mice lacking the breast-cancer transgene. In the second stage, we developed a metric to learn whether women with breast cancer manifested specific TCR sequences also present in mice developing breast cancer.

## Results

### Tumor-associated Public CDR3 amino acid sequences are a feature of the TCRα and TCRβ repertoires of mice developing tumors

**Fig 1:**
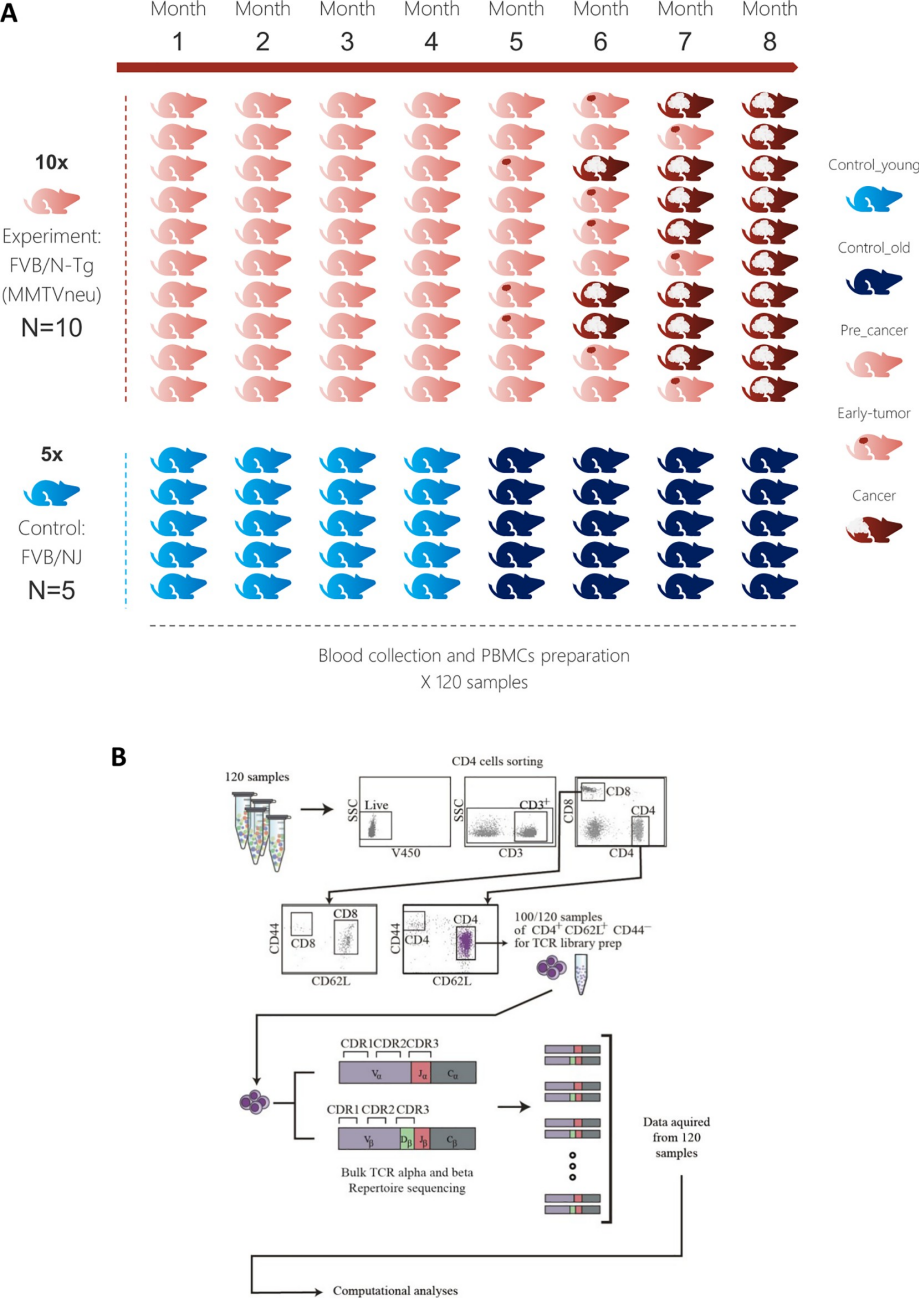
Experimental procedure. **(A)** 120 blood samples were drawn from the retro-orbital sinus of 10 FVB/N-Tg(MMTVneu), a mouse model of HER2 human breast cancer mice, and from 5 FVB/NJ Control mice. Over these 8 time-points, none of the Control mice (blue) developed any tumors. We will use the term "Transgenic" for FVB/N-Tg(MMTVneu) 202 Mul/J and "Control" for FVB/NJ strain. Progress of tumor in the ten Transgenic mice is demonstrated using the red colored samples in the figure. The last time point before tumors are shown was defined as pre-cancer and marked light red. **(B)** At each time point, the peripheral blood mononuclear cells were isolated and stained for flow cytometry. Cells were analyzed and gated for sorting using a FACS ARIA III sorter, and the CD4^+^CD62L^hi^CD44^lo^ naïve population was separated for RNA extraction and T cell receptor library preparation (see Methods). TCR alpha and TCR beta were obtained separately and not via single-cell TCR sequencing (alpha-beta pairing is not possible).

We sequenced CDR3 repertoires in the peripheral blood of 10 FVB/N-Tg(MMTVneu), a mouse model of HER2 human breast cancer mice, and from 5 FVB/NJ Control mice (Fig 1). We will use the term "Transgenic" for FVB/N-Tg(MMTVneu) 202 Mul/J and "Control" for the FVB/NJ strain. The group of Transgenic mice developed tumors (see Fig 1). In the text, we refer to these mice as tumor-developing or, alternatively, as Transgenic mice, according to context. We focused on the largest recoverable subset of T cells, those bearing the markers CD4^+^CD62L^hi^CD44^lo^, which comprises 65% to 85% of the T cells in a blood samples. These T cells are of the naïve compartment. While some literature discusses subsets of this compartment, which include various sub populations, including some that have been referred to as atypical naïve–like memory/effector cells [6], the vast majority of these cells are naïve. Some recent works show that the unique TCR reactivity to immunogenic pathogen-derived epitopes is present already in the naíve repertoire prior to immune expansion in B6 mice [7] and that Naïve T cells can manifest shifting to Teffs, which can effectively kill tumor cells [8]. Interestingly, recent evidence has shown that CD4+ naive T cells significantly more heterogeneous than was previously thought, and that these sub populations of T cells are diverse in phenotypes, differentiation stages, persistence, functions, and anatomic localizations [6].

**Fig 2:**
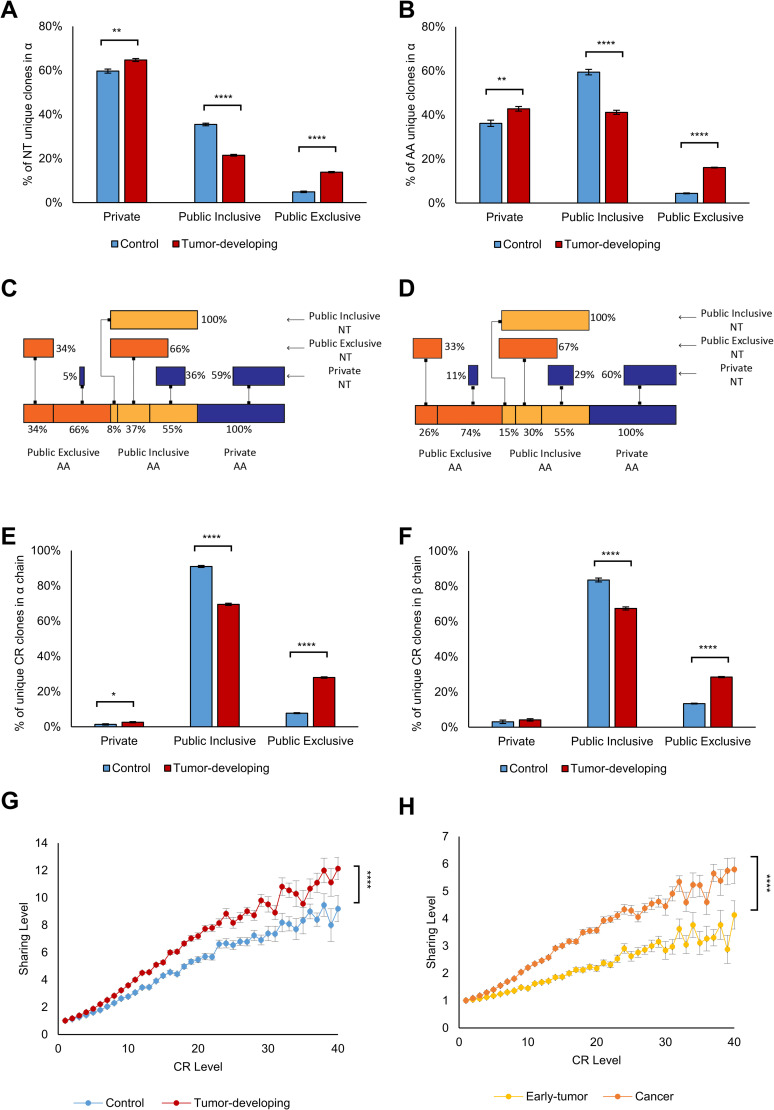
Convergent Recombination Dominates the Public Repertoire and the tumor-developing mice repertoire. Public repertoires and their subtypes are shown in tumor-developing mice and in Control mice using nucleotide sequences **(A)** and using AA sequences **(B)** in α chain. The Y-axis in the panels shows the percentages of “unique” clones, where we used the common definition of “unique” sequences as that in which we count each sequence only once and disregard its copy-number. The two panels show the repertoire in the α chain, but a similar effect is seen in the beta chain (S1 Fig). The different categories included in the panel bars are relative abundance of the different categories. That is, together they represent 100% of the sequences. Therefore, the color bars together sum up to 100%. **(C, D)** Convergent recombination in Control and Transgenic mice in α chain. The upper bars indicate nucleotide (NT) sequences and their division to the different Public groups (Private, Public-Inclusive and public-Exclusive), while the lower bars indicate amino-acid (AA) sequences in Control mice (C) and tumor-developing mice (D). The lines between NT and AA represent the effect we see in convergent recombination–in which different NT sequences encode to the same AA sequence and change the Public/Private balance. For example, in panel C, we can see that 5% of the NT sequences that were Private in an NT view, became 66% of the Public Exclusive when we looked in an AA view. **(E, F)** Frequencies of CR clones in the Public repertoire in α and β chains. **(G, H)** Correlation between the averaged mouse-mouse sharing level and CR level in Control versus tumor-developing groups **(G)** and in the Early-tumor group versus Cancer group **(H)** in β chain.

To minimize the possible bias that accompanies the use of multiple groups and diverse sample sizes, we combined samples from all the time-points of a mouse and subsampled equal numbers of sequences in 5 mice each of the test and Control sets: 843,326 CDR3 β chain sequences and 116,751 α chain sequences (see Methods). We first filtered for Public AA sequences, which are CDR3 AA sequences appearing in at least two different mice. Public CDR3 types are termed Inclusive if they appear in both Control mice and tumor-bearing Transgenic mice (FVB/N-Tg(MMTVneu) 202 Mul/J). Exclusive CDR3 AA sequences are shared only by mice bearing tumors or only by tumor-free Control mice. We found that Exclusive Public CDR3 types in both the α and β chains are more prevalent in the tumor-developing group than in the Control group (α chain: 14% and 5% respectively; Figs 2A, S1A and S1B; t-test: p <7.4E-08; S1 Table; β chain: 8% and 5% respectively; S1C–S1F Fig; t-test: p < 0.0097; S2 Table). In other words, tumor-associated Public sequences mark the tumor-developing mice. Interestingly, Public CDR3 types occupy between 35–40% of the sequences in all the mice. This level of sharing is consistent with the sharing levels reported in [4]. Note that we found no significant differences between tumor-developing and Control groups in repertoire unique clones, diversity, clonality or CDR3 length distributions (S2 Fig).

The metric by which we are looking at the (dis)similarities first aims to see if the complete set of mice share higher similarities during the early time points. We calculated overlaps between samples, by following these steps: (1) We defined samples from young mice as samples from time points 1–4, and samples from old mice as samples from time points 5–8. (2) We calculated Morisita’s overlap index for each pair of samples. (3) For each combination of groups, we averaged all the pairs related to this group. For example, for the combination of ‘Control Young’ and ‘Transgenic Young’, we averaged over all pairs of samples where one sample belongs to the ‘Control Young’ group, and the other sample belongs to the ‘Transgenic Young’ group. As shown in S3A Fig, the ‘Control Young’ and ‘Transgenic Young’ groups indeed display higher similarities compared to the ‘Control Old’ and ‘Transgenic Old’ groups.

Although we screened relatively small numbers of mice, those developing tumors clearly manifested Exclusive Public CDR3 types; a smaller proportion of Control mice manifested Exclusive Public CDR3 types not found in the mice developing tumors.

### Convergent recombination (CR) dominates Public CDR3 repertoires

We measured CR levels over time in the tumor and Control mice. For each CDR3 AA sequence, we calculated the number of different NT sequences encoding that AA sequence in the individual mice, that is, each AA sequence has a “CR level” value, which is the number of different NT sequences encoded to that sequence in our subsampled data. CDR3 sequences may end up identical despite different VJ origins. That is, in our workflow, a single CDR3 could be the result of multiple, different, VJ pairs. We found a somewhat higher frequency of CR in mice developing tumors compared to Control mice in both the α chain (47% and 40%, respectively; t-test; p < 0.016; S4A Fig) and the β chain (37% and 33%, respectively; t-test; p < 0.011; S4B Fig). We also found that the α chain manifests higher CR levels compared to the β chain.

These results confirm that Public CDR3 AA sequences manifest greater CR levels than do Private sequences [9]. Fig 2A and 2B show that CR in the Public-Inclusive groups is double that of the Private CDR3 types. Fig 2C and 2D show that the Public tumor-associated CDR3 (publicness is determined according to AA sequence) types originate from a smaller portion of the Private types (determined according to NT sequence) in the Control group compared to the Tumor group (5% and 11%, respectively). Moreover, non-CR CDR3 types dominate the Private repertoires (S4C and S4D Fig). In addition, we identified a significant contribution of CR to the Public TCR repertoire– 98% of the CR types in the α chain are Public (Fig 2E) compared to 22%-40% of the non-CR types (S4C Fig). The same phenomenon was observed in β chain– 96%-97% of the CR CDR3 types were Public (Fig 2F), in comparison to 16%-28% of the non-CR types (S4D Fig). We compared the tumor-Exclusive frequency to the Control-Exclusive frequency. We calculated the levels of CR for each group of Exclusive clones. As shown in S4E Fig, the averaged CR level in the tumor-developing group is significantly higher than the averaged CR level found in the Control group (t-test: p<0.0001).

Finally, we observed evolution of CR and publicness over time. We quantified CR levels linked to publicness in the following groups of mice: Controls, Transgenic mice before palpable tumors, Transgenic mice with early-tumors and Transgenic mice with large tumors. Fig 2G and 2H show that CR levels increase in tandem with the degree of the averaged publicness in all the groups. However, the connection between CR and Public sharing is specific to the group. In the tumor-developing group, compared to the Control group, Public sharing increased as CR increased (lr test: p<2.2e-16; Fig 2G). This suggests that selection of specific CDR3 types is dominant in Transgenic mice. Separating the Transgenic group into early-tumor and cancer samples, we observed that sharing was greater in the cancer group than in the early-tumor group (lr test: p<2.2e-16; Fig 2H); thus, high degrees of CR and highly shared between mice, Public sequences develop early, even before the emergence of palpable tumors.

### Human patients and tumor-developing mice express many of the same Public-Exclusive clones

**Fig 3:**
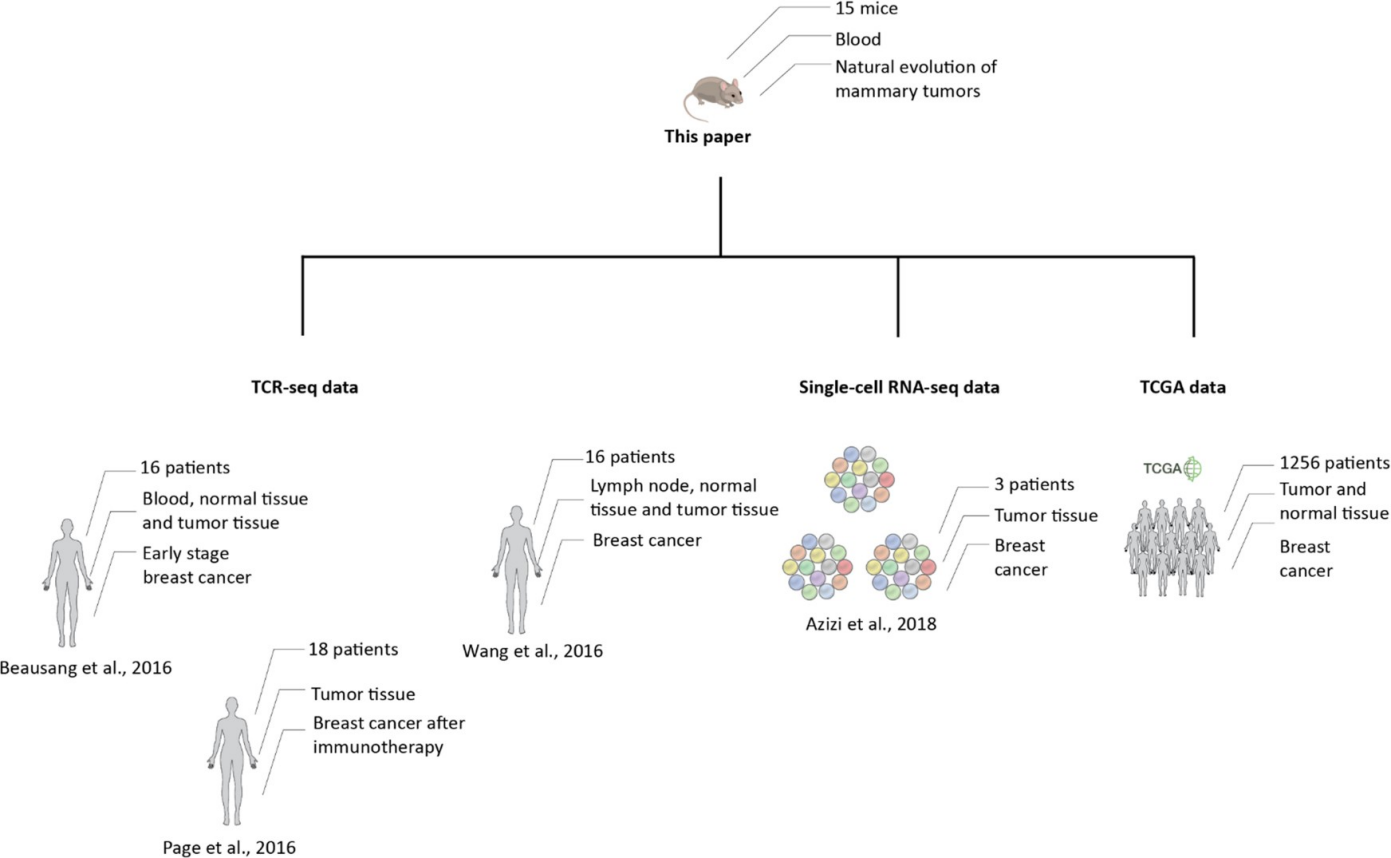
Comparing human T cell repertoire data and mouse T cell repertoire data. To learn more about the connection between the repertoires of mouse mammary tumor and human breast cancer, we studied the three different types of datasets (left to right): (1) TCR β-seq data from 50 breast cancer patients from 3 different studies, in which different conditions and different tissues were studied. (2) Single-cell RNA-seq TCR data of 3 breast cancer patients obtained from Azizi et al. (3) TCR sequences extracted from RNA-seq data of breast cancer patients obtained from The Cancer Genome Atlas (TCGA).

We studied the TCRβ-CDR3 repertoire of three human sources: 1) TCR sequences that we extracted from RNA-seq data of breast cancer patients obtained from The Cancer Genome Atlas (TCGA) [10]; 2) bulk TCR-seq data from previously published human TCR breast cancer datasets: Wang et al [11], Page et al [12] and Beausang et al [13]; and 3) Single-cell RNA-seq TCR data of 3 breast cancer patients obtained from Azizi et al [14] (Fig 3). This analysis, detailed below, supported a number of conclusions: 1) humans with breast cancer share Public CDR3 AA sequences with mice developing spontaneous breast cancer; 2) cross-species CDR3 AA sequences rank similarly in mice and human patients, detected by our ranking metric; and 3) Public, cross-species CDR3 AA sequences arise from NT recombinations that do not overlap in humans and mice–in other words, mice and humans manifest identical CDR3 AA sequences, but the NT recombinations that each species uses to generate these shared AA sequences are completely different.

As we detail below, mice and humans with breast cancer share TCR CDR3 AA sequences. We compared the mouse data we produce in this work with data from human samples and extracted different subsets of CDR3 sequences detailed in the Methods section. The first subset of cross-species sequences included the set of CDR3s from this work and the set of CDR3s from the 3 human-data papers mentioned above. We found 7513 cross-species sequences in this subset. S5 Fig shows a comparison of mouse-specific NT and AA sequences (inner circle) with cross-species mouse sequences shared with human patients (outer circle); the groups were divided into Private, Public Inclusive and Public Exclusive as previously described. The lower two wheels in the figure describe NT sequences. We can see that the tumor-developing mice express more Public Exclusive and less Public Inclusive sequences than the Control mice. AA sequences, in contrast, show a significantly greater proportion of Public Inclusive sequences than Public Exclusive sequences. Nevertheless, tumor-developing mice do express relatively more Public Exclusive sequences. Thus, the NT view and the AA view portray different perspectives, and mice developing tumors show more Public Exclusive sequences of both NT and AA types.

**Fig 4:**
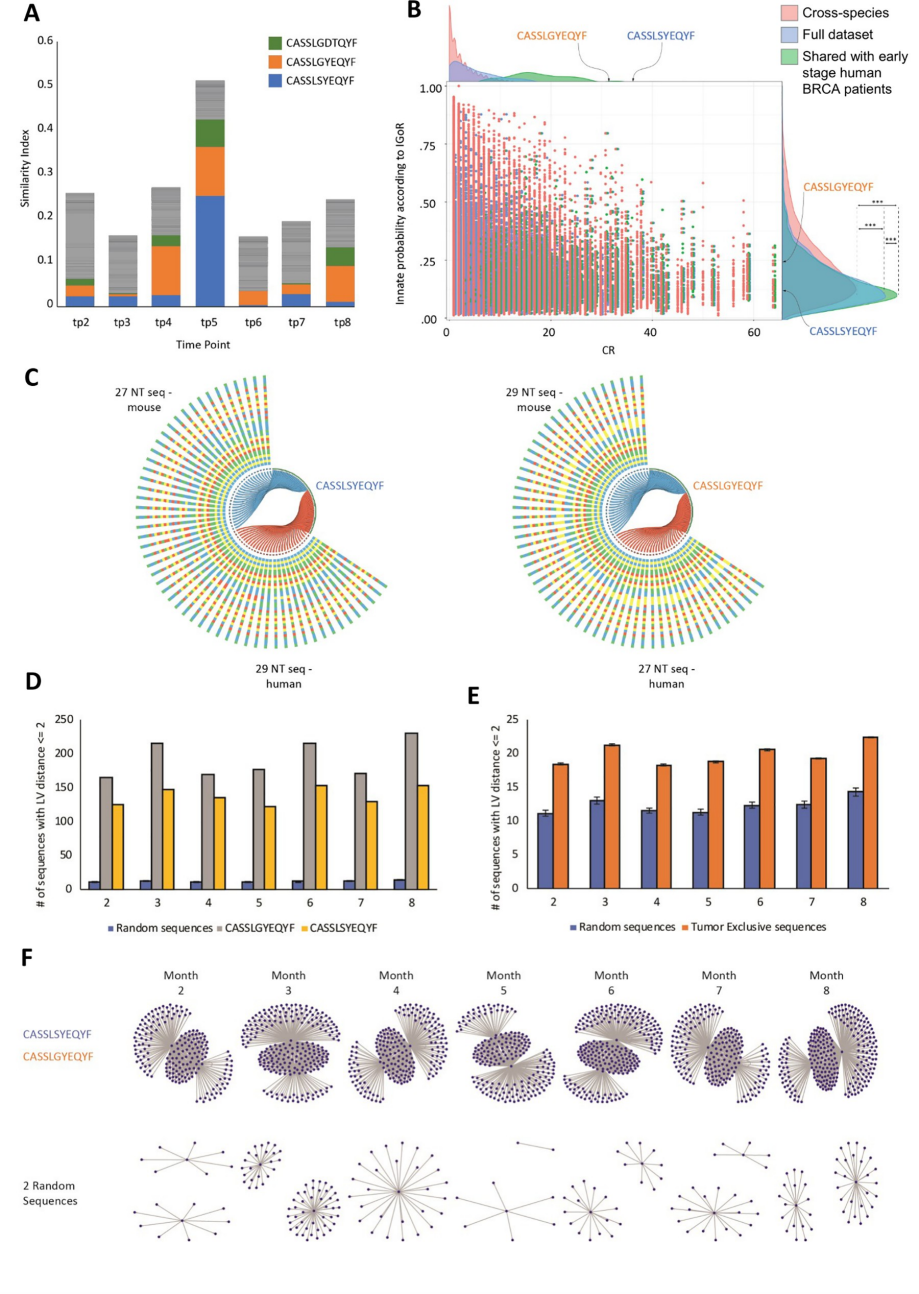
Specific Cross-species TCRβ Clones Dominate the tumor-developing Group. **(A)** We ranked the 258 cross-species sequences according to their abundance in each sample. To visualize similarities between the ranking in each time point and the samples from early-stage breast cancer patients as in Beausang et al., we stacked bars from each of the 258 ranking lines. The area of each bar has been determined so that it is reciprocal to (ranking in human X ranking in mouse). In that manner, if, in a specific time point, a clonotype is ranked #1 in mouse samples, and is also ranked #1 in human samples, it would demonstrate the largest area. Color of sequences is preserved across bars, so that we see that three sequences dominate the similarity between samples: blue, orange and green. These sequences are included in Table 1, and, as the Table further indicates, have been previously associated with Melanoma, with Influenza, and with Diabetes. In this panel we collectively used the grey color to indicate all the sequences that were not one of the three sequences colored differently in the panel. **(B)** We used IGoR (38) (see Text) to learn of any differences between the populations of cross-species sequences used in panels and the full collection of sequences. To do that, we plotted each sequence as a dot on the graph. Red dots represent cross-species sequences, blue dots represent the full set of all sequences, and green dots represent the 4700 NT sequences that code for the 258 AA sequences that are shared across all time-points in our mouse samples and with human samples. The vertical location of the dot is determined by its IGoR value and the horizontal location by its CR value. The right-hand side curves present the histogram over the IGoR values, and the upper-side curves present the histograms over CR values. We used a Kolmogorov–Smirnov test to estimate p-value for the differences between distributions. Indeed, a highly significant p-value (p<2.2x10^-16^) has been obtained, that demonstrates a large difference between the sequence populations. It is interesting to note, as shown by the three CR histograms on the upper side of the panel, that the CR values of these three populations of sequences also come from extremely different distributions (p<2.2x10^-16^). We also highlighted the locations of the two sequences that are described in panel A. **(C)** The two highly ranked clones in the cross-species analyses are visualized for their CR sources. In the panel, each nucleotide sequence is connected to its translated AA sequence. Edges are blue if they originate from a mouse sequence and red if they originate from a human sequence. The colored bars represent the NT sequences encoded to these AA sequences. Each color represents different nucleotide: T–blue; G–yellow; C–green; A–red. As the panel shows, there is no overlap between sources for these two AA sequences. **(D)** The number of sequences that differ from CASSLGYEQYF (grey bars), CASSLSYEQYF (yellow bars) and from 1000 random sequences (blue bars), by 1 or 2 AA. The X-axis provides the different time points obtained from our mouse data, and the Y-axis represents the number of similar sequences with Levenshtein edit distance of up to 2. **(E)** A representation of the sequences that are close in their edit distance to tumor-associated sequences (orange bars) and to 1000 random sequences (blue bars). **(F)** Network representation of similar sequences to CASSLGYEQYF and CASSLSYEQYF (upper networks), and to 2 random sequences (lower networks) over time.

We compared the mouse data to samples from early-stage breast cancer patients from Beausang et al., and found 258 sequences that were shared between these human samples and the collection of time points across our mouse data (see Methods). To quantify these 258 CDR3 cross-species sequences, we produced the bar-chart shown in Fig 4A. Every time-point in the bar-chart contains a stack of 258 bars. The area each bar occupies in the stack is inversely proportional to an area defined by the ranking of the sequence at the specific time-point, multiplied by the ranking of the sequence in the human samples, such that similarly ranked sequences occupy a larger area. According to Fig 4A, time-point 5 (which is associated with early-tumors in mice) is the time-point most similar to early-tumors in patients. We can also see that a small set of sequences (blue (CASSLSYEQYF), orange (CASSLGYEQYF), and green (CASSLGDTQYF) in the bar-chart) dominate the chart. The CASSLSYEQYF and CASSLGYEQYF CDR3 sequences are ubiquitous to TCR repertoire studies, and have been associated with Melanoma and Influenza (Table 1). Recent findings from other studies show associations to these phenotypes [15,16]. Additionally, 829 of the 14,349 (6%) tumor-associated CDR3 sequences found previously, were also tumor-associated in human samples; that is, they were found in tumor samples but not in normal samples.

**Table 1:** CDR3 sequences that show cross-species, tumor-Exclusive, behaviors.

CDR3 sequence	VDJdb	Peptide in VDJdb	McPAS-TCR	Peptide in McPAS-TCR	References	Ranking
CASSYSYEQYF	-	-	Melanoma	-	[17]	1
CASSPTGYEQYF	-	-	-	-	[17,18]	2
CASSLGYEQYF	-	ELAGIGILTV	Influenza	LPRRSGAAGA	[12,19–21]	3
CASSLSYEQYF	-	-	Influenza	LPRRSGAAGA	[17,22]	4

To characterize the differences between the specific groups of cross-species sequences and the general population of CDR3 sequences, we utilized IGoR (Inference and Generation Of Repertoires) [23], which is a tool that probabilistically annotates sequences. It takes B- or T-cell receptor sequence reads and quantitatively characterizes the statistics of receptor generation. Fig 4B shows the results over the three groups discussed here (see Methods): the collection of all sequences, the collection of all cross-species sequences (7513 AA sequences that were found in both mouse data and the 3 human datasets), and the collection of the unique set of 258 cross-species sequences that were inherent to all samples, together with human samples. As the figure shows, the three curves are significantly different from each other. Moreover, cross-species sequences and the set of 258 sequences were associated both with a distribution of relatively low probabilities and with high CR distributions, displayed at the top of the panel. These findings indicate that, even though the sequences were not produced at higher probabilities, they were selected to appear as such. Of special interest, and highlighted in the figure, are two sequences–CASSLGYEQYF and CASSLSYEQYF–which, in spite of their single amino acid difference, appear with very different IGoR scores and with very high CR values (notice that we did not include the third sequence CASSLGDTQYF as part of the analyses, since it did not produce as meaningful findings as the other two sequences).

When we examined the tumor-associated sequences that were shared between our mouse data and the human data subset of 7513 cross-species sequences, we identified 639 tumor-associated cross-species sequences. To examine the gene usage origin of these sequences, we analyzed V, J gene usage. We show the heatmaps in S6 Fig, which represent the correlations between the different V and J usage in tumor-associated cross-species clones. As shown, there are multiple Vs that encode these sequences, some of them are close across species, such as TRBV31 in mouse and TCRBV30 in human, and some are close within species, such as TRBV5 and TRBV2 in mouse. As for J gene usage, there are closer orthologues across species.

**Fig 5:**
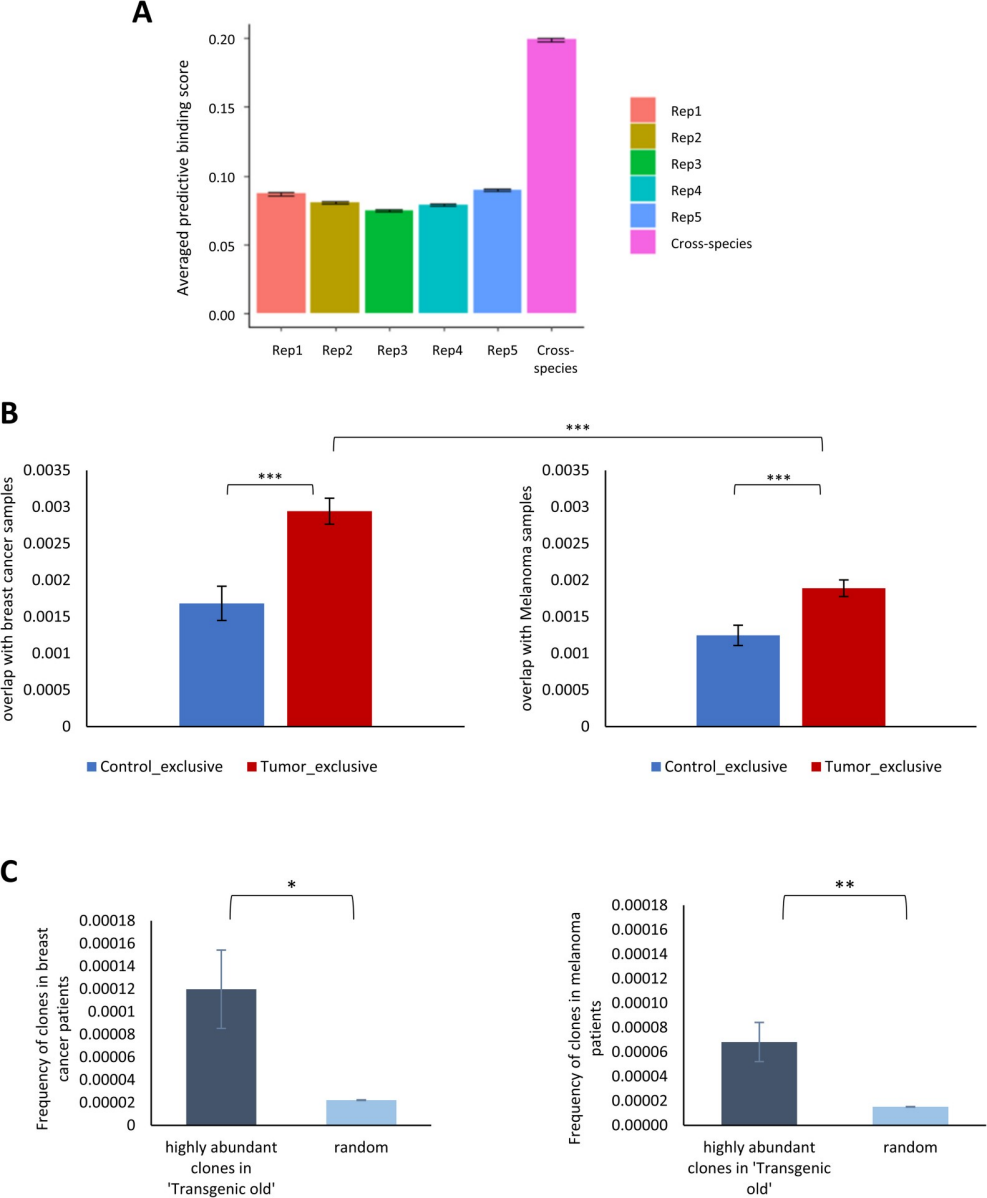
Cross-species tumor-associated clones. **(A)** The averaged predictive binding score to tumor peptides of tumor-associated cross-species sequences (right bar) and 5 random subsets of sequences. **(B)** The averaged Jaccard overlap index between control-exclusive sequences and tumor exclusive sequences to breast cancer samples (left plot) and Melanoma samples (right plot). **(C)** The frequency of highly abundant sequences from ‘Transgenic Old’ samples and random sequences in breast cancer samples (left plot) and melanoma samples (right plot).

To see if the TCR sequences we singled out as tumor associated through our mouse-human workflow are associated with human tumors, we analyzed the 258 cross-species tumor sequences over a set of tumor-peptides, from the McPAS database [24], that are known (from the literature used to build the dataset) to be associated with human tumors. To perform this analysis, we used ERGO, a deep-learning based tool for the prediction of TCR-peptide binding [25]. From MCPAS, we selected for the peptides that are tagged with a cancer classification (n = 257). We then created all possible CDR3-peptide pairs of these cancer-peptides and our 258 cross-species tumor sequences (n = 258x257 = 66,306 pairs). We used ERGO to find the predictive binding score for each CDR3-peptide pair. To see the significance of these scores, we compared them with a set of randomly chosen 258 CDR3 sequences from our data. We applied the same steps to these randomly selected TCR sequences as we did for the 258 cross-species TCR tumor associated sequences. To see the significance of these findings, we repeated the process (random pooling followed by ERGO) five times. Fig 5A provides the results of the process and the average predictive binding scores. As the figure shows, the cross-species tumor sequences pairing with human tumor peptides is significantly higher than the other pairs of CDR3-tumor peptides.

To further examine the significance of the tumor-exclusive clones, we compared the sharing between control-exclusive and tumor-exclusive clones in mouse to human breast cancer dataset (Beausang et al.) and to non-breast cancer dataset (Robert et al.; Melanoma). We subsampled an equal number of sequences from all of the examined samples, both mouse and human samples (n = 27776), and calculated the sharing between every 2 mouse-human samples. The sharing is defined as the Jaccard overlap index with a modification that takes into account the frequency of clones [26]. Fig 5B shows that the tumor-exclusive clones have a significantly higher overlap with human cancer patients compared to control-exclusive in both breast cancer human patients (p>3.2E-05) and human Melanoma patients (p>1.7E-04). Additionally, when we compared the overlap of tumor-exclusive with the 2 types of cancer, we found significantly higher mouse-human sharing with breast cancer compared to melanoma (p>1.6E-06).

To look at the difference between ‘Transgenic Young’ and ‘Transgenic Old’ samples, we compared the highly abundant sequences from these two groups with sequences we obtained from two human cohorts. First, we identified a set of highly abundant sequences in mouse samples by collecting the CDR3 sequences that appeared both in at least 10 “Transgenic Young” samples and in at least 10 “Transgenic Old” samples. This initial filtering resulted in a set of 345 sequences.

To see which of these sequenced is highly abundant in the “Transgenic Old” samples, we used a t-test, with which we identified 13 such sequences with significant differences in their copy numbers between the two groups. We then used these sequences to see their copy numbers in a cohort of TCR sequences from breast cancer patients and in a cohort of TCR sequences from Melanoma patients. For a comparison with other sequences, we repeated the above steps 1000 times using random choices of 13 sequences (out of the 345 sequences). As can be seen in Fig 5C, the frequency of the clones that were highly abundant in the “Transgenic Old’ is significantly higher compared to the random clones both in samples from breast cancer patients (p<0.013) and in samples from melanoma patients (p<0.003). Interestingly, the frequency of the ‘Transgenic old’ highly abundant clones is higher in the breast cancer samples compared to melanoma samples.

### Public, cross-species CDR3 AA sequences in humans and mice arise from different NT recombinations

To learn about the nucleotide source of the specific sequences we identified to be simultaneously high ranking in mouse and human samples (blue and orange in Fig 4A and 4B), we compared the NT sequences that generated these AA sequences. The results are shown in Fig 4C and 4D, where we connected each NT sequence to its end-result AA sequence. Edges are blue if they originate from a mouse NT sequence and red if they originate from a human NT sequence. As the figure shows, the same AA sequence originates from multiple NT sequences. However, the NT sequences show no overlap between mouse and human samples. The AA sequence in Fig 4C is the result of 27 different NT sequence recombinations in mice and 29 different NT sequences in humans. The AA sequence in Fig 4D originates from 29 different NT sequences in mice and 27 different NT sequences in humans. Whatever the upstream NT recombination, the CR process strongly selects for a specific AA sequence. S7 Fig shows the NT display of the AA compositions presented in Fig 4C. The figure reveals the recombination effect: the same AA sequences were built on a combination of 9 different TRBVs (-2,3,4,5,19,21,24,26,29), while TRBJ2-7, both for mouse and for human, was used. The colored bars represent the NT sequences encoding to these AA sequences.

To further research the importance of the two AA sequences CASSLGYEQYF and CASSLSYEQYF, we calculated the numbers of other sequences very similar to them in a network configuration [27,28]. Here, we define similar sequences as AA sequences with an edit distance (Levenshtein distance) of up to 2. We created a pool of all the sequences from the tumor-developing group for each time point, and then only used the top 10,000 sequences–in order to avoid a bias stemming from different sample sizes. We then counted the number of sequences close to the two given AA sequences, and compared these results with 1000 random sequences. Fig 4D shows that the number of sequences differing from CASSLGYEQYF and to CASSLSYEQYF by 1 or 2 AA is significantly higher than the average number of sequences equally similar to any of 1000 random sequences. We can also see that this organization does not significantly change over time. Similar behavior is observable in the tumor-associated sequences (Fig 4E). Fig 4F shows the differences between the distance networks of CASSLGYEQYF and CASSLSYEQYF and two random sequences. Each node represents a similar sequence to these two sequences. We can see that the networks of CASSLGYEQYF and CASSLSYEQYF are significantly larger than those of the random sequences.

### The relationship between TCR clones and different breast cancer stages

To learn more about the connection between the CDR3 types we identified in the mouse model and those from human patients, we analyzed RNA-seq samples from breast cancer patients that were part of the TCGA cohort. Using data from genome alignment and TCR alignment on the 1,256 breast cancer samples, we extracted roughly 60 α/β sequences per sample with a total number of 38,068 α NT sequences and 30,524 β NT sequences. Out of these sequences, 874 AA β sequences and 300 AA α sequences were also found in our mouse subsampled data.

**Fig 6:**
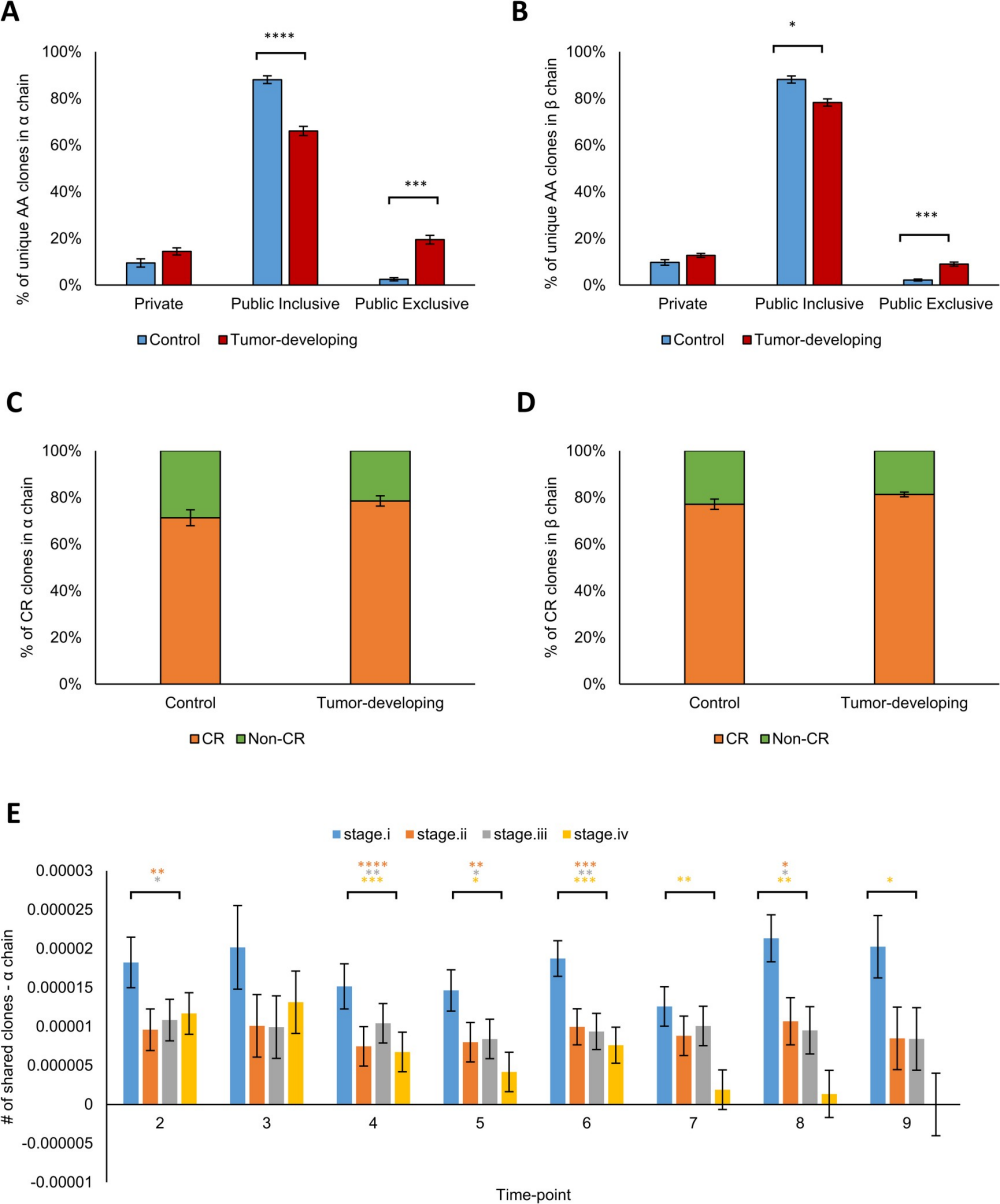
The relationship between TCR clones and different breast cancer stages. Public repertoires of the TCGA-associated TCR clones and their subtypes are shown in tumor-developing mice and in Control mice for α chain **(A)** and β chain **(B)**. Frequency of Convergent recombined clones shared between TCGA samples and mouse samples in α **(C)** and β chain **(D)**. **(E)** Frequency of shared clones between TCGA samples and tumor-developing mice in different stages of breast cancer in α chain clones. For each sample, we calculated the number of shared clones according to the following formula: (Shared clones between sample and stage) / (# of clones in a sample x # of total cases of a stage).

When examining the different Public groups of the sequences shared with our mouse samples and TCGA samples, we found that the sequences from the tumor-developing group share significantly more Public Exclusive clones compared to the Control group in both the α chain (20% and 3%, respectively; Fig 6A; t-test: p< 0.0003) and β chain (9% and 2%, respectively; Fig 6B; t-test–p< 0.0005). In addition, the percent of CR clones in this subset of cross-species CDR3 types is significantly higher than that observed in our data for both α and β chains (71%-81% and 37%-63%, respectively; Figs 6C, 6D, S4A and S4B).

TCGA breast cancer data includes clinical information about each patient, including the stage of the disease. We compared TCGA disease stage data to our longitudinal time point data (see Methods). For each time point we calculated the similarity between sample and stage using the following formula: (Shared clones between sample and stage) / (# of clones in a sample x # of total cases of a stage). S3 Table shows the number of mouse-human-shared clones between each sample and each stage. The analysis indicated that across all mouse time points in the α chain, the greatest similarity is between mouse sequences and stage 1 breast cancer. Least similar is stage 4 breast cancer (Fig 6E). The same is seen in β chain data (S8 Fig). These results might be influenced by the source of T cells: The TCGA data come from the tumor sample itself, but the mouse data come from blood samples. The findings might suggest that T cells from later stages of tumor development are not present in peripheral blood or, that the similarity between mouse and human CDR3 sequences is limited to early stages of breast cancer. It is also conceivable that cancer treatments might lead to major modifications of the T-cell repertoire, absent from the mouse population.

### Tumor-developing mice show higher similarity to human breast-cancer patients than do Control mice

Single-cell data from T cells can provide a more refined resolution than bulk populations; it is possible to match α and β sequences and to study whole transcriptomes. To gain a better view of the TCR repertoire in human breast-cancer patients and its relevance to the longitudinal mouse study, we analyzed single-cell data available from Azizi et al. [14], who sequenced tumor tissue from 3 patients with breast cancer using 10x single-cell RNA-seq sequencing. We identified the set of α and β sequences that overlapped with our mouse data and found the α-β pair of each cell. This resulted in 3168 unique cells with 2110 different α-β pairs. Next, we searched for these α-β pairs in our data. An α-β pair was defined as shared with a mouse sample if both the α CDR3 sequence and the β CDR3 sequence were present in that sample. We found 582 α-β (single-cell) pairs whose alpha and beta chains appeared in the same sample in our mouse data.

**Fig 7:**
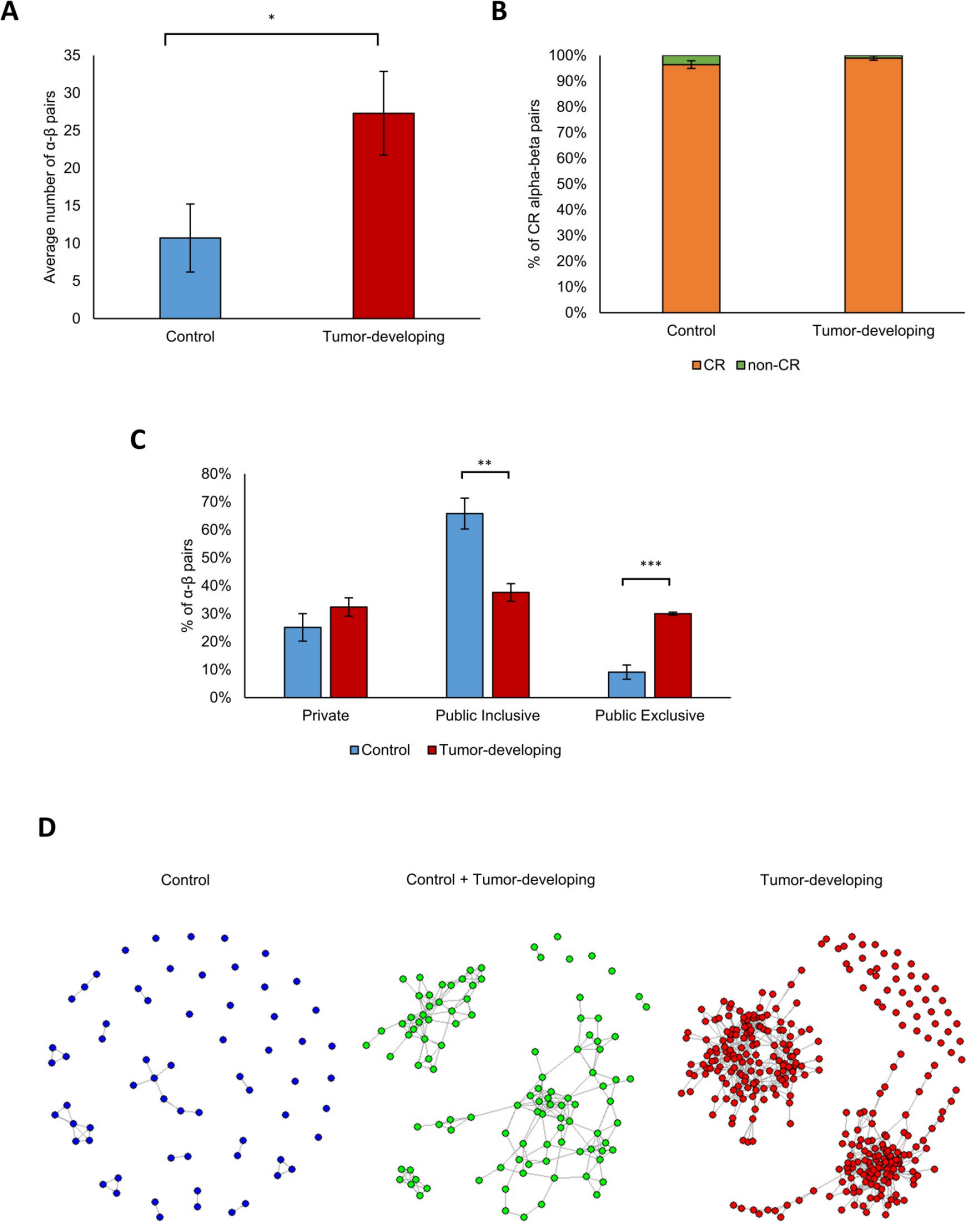
Transgenic mice share a higher similarity with human breast cancer patients than Control mice. **(A)** The number of different human α-β pairs shared with our mouse data within the Control group (blue bar) and the Transgenic group (red bar). **(B)** The percent of convergent recombined (CR) human α-β pairs in the Control and Transgenic mice. **(C)** The percent of the different Public groups (Private, Public-Inclusive and Public-Exclusive) in the Control group (blue bars) and the Transgenic group (red bars). **(D)** Distance networks of all the α-β pairs shared with our mice subsampled data that appear only in the Control group (blue network), only in the Transgenic group (red network) and in both groups (green network).

We found that the tumor-developing mice shared a significantly higher number of human α-β pairs in comparison to the Control group (27 α-β pairs per sample and 11 α-β pairs per sample, respectively; Fig 7A; t-test: p< 0.024). To learn whether CR is dominant in this subset, we averaged the CR levels of the α and β sequences. Indeed, this subset of the repertoire showed a much higher rate of CR in comparison to the total repertoire (Figs 7B and S4A–S4B). The Public repertoire presented the same effect as the CR, and was expended in both the Control and the tumor-developing mice (9% and 30%, respectively, in comparison to 7% and 12% in the total repertoire). However, the Public Exclusive repertoire was significantly higher in the tumor-developing mice compared to the Control mice (Fig 7C and 7D; 30% and 9% respectively; t-test: p < 0.0009), and compared to the total repertoire of α and β chains in tumor-developing mice (16% and 12%, respectively; Figs 2B and S1D).

Finally, we mapped single cells to the different groups. For each cell, we checked whether its α-β pair appeared in only or in both of the groups. For each group (Control, tumor-developing and shared between these 2 groups), we calculated the Levenshtein distance between the subsets of α-β pairs. In this analysis, we allowed up to 3 different AA between two pairs. As can be seen in Fig 7D, the tumor-developing network (red circles) was significantly larger than the Control network (blue circles) or the shared network (green circles); this suggests that the tumor-developing mice share significantly higher similarity with human breast-cancer patients than do the Control mice. Interestingly, we also found that the tumor-developing network can be divided into 2 main subnetworks. Combining all the human datasets, we found that the Control group dominates the subset of CDR3 types that are Public Inclusive, and that the tumor-developing group dominates those that are Public Exclusive (S9 Fig).

## Discussion

The objective of this study was to investigate whether the TCR repertoires of mice with breast cancer develop representative CDR3 sequences. We focused on the CDR3 regions of the alpha and beta chains of the TCR because the CDR3 domain binds the processed antigen epitope presented to the responding T cells [29]; thus, the CDR3 region of the TCR is responsible for antigen-specific T-cell recognition.

We initiated the study with a longitudinal survey of TCR repertoires monitored in the peripheral blood of individual mice that were in the process of spontaneously developing breast tumors; We recovered a subset of T cells based on their CD4^+^CD62L^hi^CD44^lo^ markers. CD4+ T cells are a key component of tumor immunity via their communication with several types of immune cells, direct tumor killing and by providing support to CD8+ T cells [30]. Based on molecular markers, these traits in human immunity are usually attributed to cells with an effector phenotype (CD62L^lo^CD44^hi^). In our study, the CD4+CD62L^hi^cd44^lo^ subset was the largest recoverable subset out of peripheral blood.

Sorting to the CD4^+^CD62L^hi^CD44^lo^ group still leaves multiple cell types that should be separately discussed. These include conventional CD4+ T cells that will recognize antigen peptides presented by MHC class I. These cell types, in human, might include different stages of T cell development and differentiation of naïve CD4+ cells from unprimed quiescent to effector and central memory cells [6,31] and Stem cell memory T cells [32]. Several other prominent populations of ‘unconventional’ T cells might also be included, and were analyzed and found at small frequencies in our data. We further studied data from these cell types, even though they are CD44^hi^, to exclude the possibility that they are responsible for some of the trends we saw. These T cells recognize non-peptide antigens presented by specialized monomorphic MHC class I–like molecules [33] and have unique and conserved TCR repertoires. These include CD1-restricted natural killer T cells (NKT cells) [34–36], MR1-restricted mucosal associated invariant T cells (MAIT cells) [37,38]. We found only 4 CDR3 sequences that can be classified as MAIT cells. We also identified 96 sequences in the Control group, and 201 sequences in the Transgenic group, that could be tagged as iNKT cells. However, there was no statistically significant difference in the frequency of these cells between the groups.

Another aspect of the compartments we sorted using the markers is that these are some of the T cells previously addressed as naïve, while in fact this is not a homogeneous pool of cells awaiting activation. The compartment previously addressed as naïve T cells is actually a complex mixture of cells at different developmental stages and of diverse ages [39]. Further, there are inflows of new cells from the naive pool that strongly impact memory cell [40].

In mice there are differences between RTEs (recent thymic emigrants) and mature naive T cells and between naive populations that are not replaceable and high-rate renewed cells [41].

High-throughput biology, high-resolution biology and a host of technologies are quickly adding changes to current immunological theory. The nature of memory T cell differentiation signals, the extent of their fate diversity, the lineage relationship between different fates, and the degree of plasticity at different stages of differentiation [42] are projecting on the previous stages of these cells maturation. Together with the various sub populations we mentioned earlier, we see many degree of freedom in T cell populations in spite of phenotype characteristic.

We here show that the TCR repertoires of these T cells do reflect the tumor-bearing state. Most importantly, this mouse model provided two advantages in the quest for tumor-associated CDR3 sequences: sampling inbred mice enabled us to circumvent the genomic variability inherent in outbred humans, and the longitudinal monitoring of T-cell repertoires during actual cancer development supported a level of confidence in the association of repertoire changes with the actual development of breast cancer; in contrast, a collection of repertoire snapshots from a mixed collection of women sampled at different stages of the disease would not match the precision of the controlled longitudinal mouse experiment. As one of the reviewers to this manuscript suggested, it would have been extremely useful to compare the timeline of TCR repertoire changes in these mice with an equivalent set from another Transgenic mouse. Such a set could have allowed a comparison between the changes that were caused by the tumor with changes that are caused by the transgene itself. However, we were unable to identify such a dataset, and the limit of this comparison remains. It is our opinion that the significant findings we identified with human tumor samples eliminate much of the inherent randomness that may be associated with the comparison between the repertoires.

Although the numbers of mice in the mouse study were necessarily small, the information yield was meaningful; we were able to detect and characterize 7513 CDR3 AA sequences that appeared both in the mice and in the various human populations of breast cancer patients. Most importantly, 829 of these Public sequences appeared to be Exclusive for breast-tumors in both mice and humans. The results of the present study stimulate us to think anew about the host-tumor relationship.

Our most notable finding is that the development of breast cancer selects for CDR3 regions exclusive to tumor-bearing mice, shared by both mice and humans (Fig 7D). This raises the possibility that the development of breast cancer involves the expression of particular antigens; antigen selection is the simplest way to explain cross-species CDR3 AA sequences despite the fact that each species mobilizes a different set of NT recombinations to generate exactly the same AA sequence; there was no NT overlap between the species in any of the NT recombinations leading to marked convergent recombination (Fig 4C). Thus, mouse-human-shared CDR3 AA selection must come about by way of similar antigens expressed by both mice and humans in the process of tumor development–this immune similarity takes place despite the genetic differences between inbred mice and natural human populations.

It has been taught that the TCR repertoire and tumor development are stochastic events: TCR molecules are thought to be generated by a huge number of possible NT recombinations between V-D-J gene segments augmented by NT deletions and additions at the joining segments. This process is thought to be able to produce more than 10^18^ different TCR sequences [43]. Moreover, an individual TCR antigen receptor has been reported to recognize with significant avidity upwards of a million or so peptide epitopes [44]. Consequently, the chances are vanishingly small that different mice and humans would randomly select for T cells bearing identical CDR3 binding sites, even in response to the same immunogenic epitope. It is likely that certain TCR gene segments are located in positions that favor recombination events relative to other TCR gene segments [45]. However, the effects of chromosomal positioning would not be expected to determine identical repertoire recombinations in mice and humans, which show no overlap in convergent recombination (Fig 4C).

Likewise, the development of a malignant tumor is believed to result from random combinations of many mutations in various genes–including so-called driver mutations that drive tumor development, which differs markedly from the outcome of the multitude of mutations often found in so-called normal or healthy cells [46]. The observation that mice and humans bearing diverse MHC genes can express identical TCR CDR3 amino acid sequences has a precedent in the study by (Madi, Poran et al. 2017) (Madi, Shifrut et al. 2014) [47,48] showing that different strains of mice bearing diverse MHC genes expressed identical CD-4 TCR CDR3 amino acid sequences known to recognize a single defined peptide epitope; apparently, different MHC class II molecules can present the same processed peptide to their TCR CDR3 regions. The present finding of Public, tumor-associated TCR repertoires in both mice and humans developing breast cancer hints at a uniformity in the tumorogenic process as it develops in different individuals in at least two different species. Understanding the generation of immunogenic markers shared across species is likely to deepen our understanding of pathophysiological uniformities inherent in tumor development itself. Mutations may turn a normal cell into a potentially malignant transformed cell, but a single aberrant cell is not a tumor; a growing tumor is like an organ that requires the support of a microenvironment that includes blood vessels, stromal cells, a supply of growth factors, and particular metabolic energy sources, all dynamically integrated [49]. It is conceivable that breast cancer development might require similar microenvironmental interactions in both mice and humans, which are highly structured and, therefore, quite similar. Requirements for the development of breast cancer in mice and humans, across species, may impose shared processes that stimulate shared TCR repertoires. The present investigation needs to be followed up by identification of the key immunogenic entities that drive the evolution of Public, tumor-associated TCR repertoires. At present it is not possible with any degree of certainty to infer the selecting antigen from the AA sequence of the responding CDR3 TCR; new technologies are needed. Moreover, the possible candidate antigens are many and varied: normal developmental antigens expressed aberrantly by dedifferentiated tumor cells; neo-antigens generated by mutations; regulatory molecules expressed by the activated immune system itself–ergotypic activation markers, for example; tumor-associated bacteria or viruses; epitopes of stress proteins; the list goes on and on. Some Public CDR3 specificities may be annotated by their appearance in other situations [50]. In any case, Public, tumor-associated TCR repertoires might arise from common factors needed for tumor development; the elucidation of the antigenic molecules that drive Public, tumor-associated TCR repertoires could help identify commonalities in tumor development. The challenges underlined by the present study are difficult but promising.

## Methods

### Ethics statement

Approval number: 39-10-2012, Dr. Benny Motro, the committee chairman, Bar Ilan University.

### Mice

We purchased from Jackson Laboratories transgenic mice, at the age of 1 month, expressing the inactivated rat neu (Erbb2) oncogene under the transcriptional Control of the mouse mammary tumor virus promoter (FVB/N-Tg(MMTVneu) 202 Mul/J). Female mice of this strain serve as a mouse model of mammary tumor in humans–the HER2/ Erbb2 / Neu human breast cancer model [51]; this test group included 10 female mice. The transgenic mouse model we used, FVB/N-Tg(MMTVneu)202Mul/J (Jackson laboratories) is homozygous for the MMTVneu (rat) transgene. The mice are viable and fertile. There is no phenotypic effect in males. The transgene is expressed at low levels in normal mammary epithelium, salivary gland, and lung. Higher expression is detected in tumor tissue. Focal mammary tumors first appear at 4 months, with a median incidence of 205 days (6.8 months). Both virgin and breeder mice develop tumors. Tumors arise as foci in hyperplastic, dysplastic mammary glands. Seventy-two percent of tumor-bearing mice that live to 8 months or longer develop metastatic disease to the lung.

FVB/NJ strain mice, bearing the same genetic background as the transgenic mice, but without the breast-cancer transgene, served as Controls; the Control group included 5 mice. The mice were housed under specific pathogen-free conditions in accordance with applicable laws and regulations, and approved by the responsible animal care and ethical committee. Mice were monitored by palpation for tumor development and bled monthly for up to 9 months, or until they had to be sacrificed because of tumor growth.

### T-cell isolation and testing

Peripheral blood was sampled from the retro-orbital sinus of each of 15 mice once a month for 8 time points (a total of 120 samples). Mononuclear cells were isolated by density gradient centrifugation using Ficoll (Ficoll Paque plus, GE Health Care–according to the manufacturer’s instructions). At the termination of the experiment, single-cell suspensions were prepared from the thymus and spleen of each mouse. For cell sorting, the cells were stained with the following fluorescently labeled monoclonal antibodies: anti-CD4 Pacific Blue (BD), anti-CD25 PE (eBioscience), anti-CD44 APC (BD) and anti-CD62L PE-Cy7 (eBioscience); viability was determined using the Fixable Viability stain 450 (BD Horizon). Cell sorting was performed using a FACS ARIA III sorter; CD4^+^CD62L^hi^CD44^lo^ T cells, the most prevalent T-cell population, was used for CDR3 sequencing. After sorting, the cells were pelleted and resuspended with 300μl of RNAprotect cell reagent (Qiagen). The cells were stored at minus 80°C until RNA extraction. RNA was purified from RNAprotect-stabilized cells using the RNeasy Plus Mini Kit. After RNA extraction, samples were run on TapeStation to estimate quality.

### High-throughput sequencing of the T cell repertoire

Cells were FACS sorted (by ARIA III sorter) to obtain CD4^+^CD62L^hi^CD44^lo^ T cells. RNA was prepared using Qiagen’s RNeasy kit. Isolated RNA was reverse transcribed using a biotinylated oligo dT primer. An adaptor sequence was added to the 3' end of all cDNAs, which contains the Illumina P7 universal priming site and a 17-nucleotide unique molecular identifier (UMI). Products were purified using streptavidin-coated magnetic beads followed by a primary PCR reaction using a set of the following primers:

mouse-TCRalpha

5'ACACTCTTTCCCTACACGACGCTCTTCCGATCTATCTTTTAACTGGTACACAGCAG3'

mouse-TCRbeta

5'ACACTCTTTCCCTACACGACGCTCTTCCGATCTCAAGGAGACCTTGGGTGGAG 3'

The primers were designed to target the constant regions of mouse-TCRalpha and mouse-TCRbeta. The TCRalpha/beta-specific primers contained tails corresponding to the Illumina P5 sequence. PCR products were then purified using AMPure XP beads. A secondary PCR was then performed to add the Illumina C5 clustering sequence to the end of the molecule containing the constant region. The number of secondary PCR cycles was tailored to each sample to avoid entering plateau phase, as judged by a prior quantitative PCR analysis. Final products were purified, quantified with Agilent Tapestation and pooled in equimolar proportions, followed by high-throughput paired-end sequencing on the Illumina MiSeq platform. For sequencing, the Illumina 600 cycle kit was used with the modifications that 325 cycles were used for read 1, 6 cycles for the index reads, 300 cycles for read 2 and a 20% PhiX spike-in to increase sequence diversity.

### Processing of raw sequencing data

RNA-seq raw reads were aligned and mapped to V-D-J sequences using MiXCR, a computational tool that enables the processing of big immunome data from raw sequences and output quantitated clonotypes [52]. The method performs paired-end read merging and extracts human or animal TCR clonotypes providing corrections for erroneous sequences introduced by NGS [53–55]. Using MiXCR, we obtained CDR3 sequences of α and β chains. The counts of TCRα and TCRβ sequences are presented in S4 Table.

### Subsampling of the data

To minimize any bias that might stem from diverse sample sizes, we initially combined all time points of each mouse. Next, we used the set of samples from the 5 mice of the Control group, and 5 mice from the tumor-developing group, who had the highest sequences coverage. We then subsampled equal numbers of sequences for each of these 10 mice, as the lowest number of sequences obtained for each chain: 843,326 CDR3 β chain sequences and 116,751 α chain sequences, allowing us to keep as much of the information as possible while still normalizing the data.

### Human breast cancer RNA-seq samples

We studied the TCRβ-CDR3 repertoire of three human sources: 1) TCR sequences that we extracted from RNA-seq data of breast cancer patients obtained from The Cancer Genome Atlas (TCGA) [10]; 2) bulk TCR-seq data from previously published human TCR breast cancer datasets: Wang et al [11], Page et al [12] and Beausang et al [13]; and 3) Single-cell RNA-seq TCR data of 3 breast cancer patients obtained from Azizi et al [14].

### TCGA data analysis

We downloaded RNA-seq samples publicly available from The Cancer Genome Atlas (TCGA)– 1256 tumor and normal tissues of breast cancer patients. We applied the following procedures to extract TCR repertoires: 1) We mapped reads to the human reference genome using STAR. 2) We designated as somatic recombinations only those reads that at least one of the mates did not map to the reference whole genome; a recombined V-D-J sequence cannot map completely to the genome. 3) We utilized MiXCR on all unmapped reads.

For the comparison of TCGA disease stage data to our longitudinal time point data, we used our full dataset (the entire set of clones within the sample, without subsampling). The different subtypes of stages obtained from the clinical information of each breast cancer patient was aggregated to the following stages: stage i, stage ii, stage iii and stage iv. We calculated the similarity between a sample and a stage using the following formula: (Shared clones between sample and stage) / (# of clones in a sample x # of total cases of a stage). Next, we averaged all the samples of a specific time point and a stage.

### Data from published papers

We used RNA-seq data from the following published articles: Wang et al. [11], Page et al. [12] and Beausang et al. [13]. In contrast to the bulk TCGA data, these authors carried out directed TCR repertoire sequencing.

To be able to compare the mouse data produced here and the above mentioned human data, we studied multiple subsets of sequences. These are the subsets:

1. Cross-species sequences (7513 sequences)–these are CDR3 sequences that were found in at least one sample from our mouse data and were also found in data from all three human papers described earlier.
2. CDR3 sequences shared with early-stage breast cancer patients (258 sequences)–these are sequences that were found in our mouse, tumor-developing samples across all time-points, and were also found in human early-stage breast cancer patients analyzed in Beausang et al.
3. Tumor-associated Cross-species clones (639 sequences)–these are sequences that were found in tumor samples but not in their adjacent normal samples in the human papers described earlier, and were also found to be tumor-associated in our mouse subsampled data.

### Single-cell data analysis

We developed a computational model for the assembly of CDR3 regions using single-cell RNA-seq data published by Azizi et al. [14]. The model was built using the following steps: 1) We applied MiXCR on the samples and extracted the CDR3 sequences for each cell in each patient. 2) We filtered out CDR3 sequences that did not appear in our mouse data. 3) We constructed an α-β pair of each cell barcode. 4) We filtered out all cells with more than one α or β sequence. And, 5) we searched for these α-β pairs in our mouse sequence data (after subsampling). An α-β pair was defined as shared when both the α CDR3 sequence and the β CDR3 sequence were found in our mouse samples.

### A metric to rank human-mouse cross-species CDR3 TCR clones

We developed an additional metric to compare mouse sequences with sequences obtained from the early-stage breast cancer patients reported by Beausang et al [13]. First, we identified the sequences that appeared at all the mouse time-points and in the human samples. At each time-point, we ranked the sequences according to their copy number–the highest copy number was ranked 1, the second highest ranked 2 and so on. We then ranked the human samples in the same manner as the mouse samples. The size of each clone is inversely proportional to an area defined by the ranking of the sequence at the specific time-point, multiplied by the ranking of the sequence in the human samples, such that similarly ranked sequences will have a larger size. For example, the largest area would potentially belong to a shared CDR3 sequence that is ranked #1 in both species (since 1 / ((sequence ranking in mouse = 1) x (sequence ranking in human = 1)) = 1). The smallest potentially belong to a shared CDR3 sequence ranked last in both species (1 / ((sequence ranking in mouse = 258) x (sequence ranking in human = 258)) = 0.000015).

### Versions of the different tools used in this paper

**IGOR:** Version: IGoR v1.2.0; Non-default parameters: -species mouse -chain beta—ntCDR3.

**MiXCR**: Version: MiXCR v2.1.5; Non-default parameters: -p rna-seq -s MusMusculus -OallowPartialAlignments = true.

**TCGA analysis—MiXCR**: MiXCR v3.0.3; **STAR**: STAR_2.7.0b_0206.

**Databases:** VDJdb [50] and McPas-TCR [24] were downloaded in 2018.

**Single cell analysis: MiXCR**: MiXCR v3.0.3.

**ERGO:** Parameters: Model Type = LSTM based model; Training Database = McPAS.

### Statistical tests

For the significance tests that quantify differences between means of 2 populations, we used the t-test; For the significance tests that quantify differences between 2 curves we used the lrtest function from the R package lmtest–a function that uses a likelihood ratio test to compare generalized linear models; we used the Kolmogorov–Smirnov test when we compared distributions.

Values for the error bars were calculated using the standard definition: the standard deviation divided by the square root of the number of measurements that generated the mean.

## Supporting information

S1 Table A list of all the Pubic Exclusive clones found in the tumor-developing mice in α chain. (TXT)

S2 Table A list of all the Pubic Exclusive clones found in the tumor-developing mice in β chain. (TXT)

S3 Table The number of mouse-human-shared clones between a sample and a stage obtained from the TCGA data of breast cancer patients. Rows are the different samples and columns are the different stages.
(TIF)

S4 Table The final count of productive libraries and their count of TCR α and β sequences. (TIF)

S1 Fig Public and CR repertoires. The ratios of different Public groups—Private, Public-Inclusive and Public-Exclusive repertoires in α total NT clones **(A)** and AA clones **(B)**, β unique NT clones **(C)** and AA clones **(D)** and β total NT clones **(E)** and AA clones **(F)**.
(TIF)

S2 Fig Population measures and CDR3 length distribution. **(A-D)** The number of unique sequences for the 5 different groups (Control-young, Control-old, no-cancer, pre-cancer and cancer) in α **(A)** and β **(B)** chains in an NT view and in AA view **(C, D)**. **(E, F)** Ecological diversity measures of the different groups. **(G, H)** Clonality measures of the different groups in α and β chains. **(I, J)** CDR3 length distribution of the different groups in α and β chains.
(TIF)

S3 Fig Overlaps between different groups. An heatmap of the averaged overlaps between different combinations of groups. The marked values are the averaged overlaps between Control-young and Transgenic-young, and between Control-old and Transgenic-old.
(TIF)

S4 Fig Convergent recombination. Frequency of convergent recombined clones in α **(A)** and β **(B)** and non-convergent recombined clones in α **(C)** and β **(D)**. **(E)** The averaged CR levels of the sequences found as the Control Public-Exclusive (blue bar) and in the tumor-developing Public-Exclusive (red bar) sequences.
(TIF)

S5 Fig The Public repertoire in cross-species TCR sequences. The Public repertoires in the sub-populations of clones shared between our mouse samples and human samples (7513 sequences) are shown an AA view (upper circles) and an NT view (lower circles). The inner circles are mouse-specific NT and AA sequences, and the outer circles are the cross-species mouse sequences shared with human patients. The groups are divided into Private, Public Inclusive and Public Exclusive as previously described.
(TIF)

S6 Fig V, J usage in the tumor-associated cross-species sequences. The correlations between the different Vs **(A)** and Js **(B)** usage of the tumor-associated cross-species clones.
(TIF)

S7 Fig Human and mouse NT sequences of CASSLSYEQYF. The NT display of the AA compositions demonstrated in Fig 4C. The figure reveals the recombination effect: the same AA sequences were built on a combination of 9 different TRBVs (-2,3,4,5,19,21,24,26,29), while TRBJ2-7, both for mouse and for human, was used. The colored bars represent the NT sequences encoding to these AA sequences. The colored bars represent the NT sequences encoded to these AA sequences. Each color represents different nucleotide: T–blue; G–yellow; C–green; A–red.
(TIF)

S8 Fig Frequency of mouse-human shared clones in the tumor-developing group in different stages of breast cancer in β clones. For each sample, we calculated the number of shared clones between TCGA samples and mouse samples according to the following formula: (Shared clones between sample and stage) / (# of clones in a sample x # of total cases of a stage). These numbers marked in black. The number of copies in the Control group of each intersection is marked in blue and the copies of the Transgenic group marked in red.
(TIF)

S9 Fig Cross-species shared clones between different datasets. Venn diagram representation of the number of shared clones between the different datasets from human and mouse (bulk TCR-seq, TCGA RNA-seq data, single-cell data, Control mice and Transgenic mice).
(TIF)

## References

[1] Davis MM, Bjorkman PJ. T-cell antigen receptor genes and T-cell recognition. Nature. 1988;334(6181):395–402. Epub 1988/08/04. 10.1038/334395a0 . 3043226

[2] Dupic T, Marcou Q, Walczak AM, Mora T. Genesis of the alphabeta T-cell receptor. PLoS Comput Biol. 2019;15(3):e1006874 Epub 2019/03/05. 10.1371/journal.pcbi.1006874 30830899 PMC6417744

[3] Madi A, Poran A, Shifrut E, Reich-Zeliger S, Greenstein E, Zaretsky I, et al T cell receptor repertoires of mice and humans are clustered in similarity networks around conserved public CDR3 sequences. Elife. 2017;6 Epub 2017/07/21. 10.7554/eLife.22057 28731407 PMC5553937

[4] Madi A, Shifrut E, Reich-Zeliger S, Gal H, Best K, Ndifon W, et al T-cell receptor repertoires share a restricted set of public and abundant CDR3 sequences that are associated with self-related immunity. Genome Res. 2014;24(10):1603–12. Epub 2014/07/14. 10.1101/gr.170753.113 25024161 PMC4199372

[5] Joyce JA, Fearon DT. T cell exclusion, immune privilege, and the tumor microenvironment. Science. 2015;348(6230):74–80. Epub 2015/04/04. 10.1126/science.aaa6204 . 25838376

[6] Caccamo N, Joosten SA, Ottenhoff THM, Dieli F. Atypical Human Effector/Memory CD4. Front Immunol. 2018;9:2832 Epub 2018/12/03. 10.3389/fimmu.2018.02832 30559746 PMC6287111

[7] Assmus LM, Guan J, Wu T, Farenc C, Sng XYX, Zareie P, et al Overlapping Peptides Elicit Distinct CD8. J Immunol. 2020;205(7):1731–42. Epub 2020/08/31. 10.4049/jimmunol.2000689 32868409 PMC7511415

[8] Yin Z, Bai L, Li W, Zeng T, Tian H, Cui J. Targeting T cell metabolism in the tumor microenvironment: an anti-cancer therapeutic strategy. J Exp Clin Cancer Res. 2019;38(1):403 Epub 2019/09/13. 10.1186/s13046-019-1409-3 31519198 PMC6743108

[9] Venturi V, Kedzierska K, Price DA, Doherty PC, Douek DC, Turner SJ, et al Sharing of T cell receptors in antigen-specific responses is driven by convergent recombination. Proc Natl Acad Sci U S A. 2006;103(49):18691–6. Epub 2006/11/30. 10.1073/pnas.0608907103 17130450 PMC1693724

[10] Tomczak K, Czerwinska P, Wiznerowicz M. The Cancer Genome Atlas (TCGA): an immeasurable source of knowledge. Contemp Oncol (Pozn). 2015;19(1A):A68–77. Epub 2015/02/19. 10.5114/wo.2014.47136 25691825 PMC4322527

[11] Wang T, Wang C, Wu J, He C, Zhang W, Liu J, et al The Different T-cell Receptor Repertoires in Breast Cancer Tumors, Draining Lymph Nodes, and Adjacent Tissues. Cancer Immunol Res. 2017;5(2):148–56. Epub 2017/01/01. 10.1158/2326-6066.CIR-16-0107 . 28039161

[12] Page DB, Yuan J, Redmond D, Wen YH, Durack JC, Emerson R, et al Deep Sequencing of T-cell Receptor DNA as a Biomarker of Clonally Expanded TILs in Breast Cancer after Immunotherapy. Cancer Immunol Res. 2016;4(10):835–44. Epub 2016/09/03. 10.1158/2326-6066.CIR-16-0013 27587469 PMC5064839

[13] Beausang JF, Wheeler AJ, Chan NH, Hanft VR, Dirbas FM, Jeffrey SS, et al T cell receptor sequencing of early-stage breast cancer tumors identifies altered clonal structure of the T cell repertoire. Proc Natl Acad Sci U S A. 2017;114(48):E10409–E17. Epub 2017/11/16. 10.1073/pnas.1713863114 29138313 PMC5715779

[14] Azizi E, Carr AJ, Plitas G, Cornish AE, Konopacki C, Prabhakaran S, et al Single-Cell Map of Diverse Immune Phenotypes in the Breast Tumor Microenvironment. Cell. 2018;174(5):1293–308 e36. Epub 2018/07/03. 10.1016/j.cell.2018.05.060 29961579 PMC6348010

[15] Laubli H, Balmelli C, Kaufmann L, Stanczak M, Syedbasha M, Vogt D, et al Influenza vaccination of cancer patients during PD-1 blockade induces serological protection but may raise the risk for immune-related adverse events. J Immunother Cancer. 2018;6(1):40 Epub 2018/05/24. 10.1186/s40425-018-0353-7 29789020 PMC5964701

[16] Simoni Y, Becht E, Fehlings M, Loh CY, Koo SL, Teng KWW, et al Bystander CD8(+) T cells are abundant and phenotypically distinct in human tumour infiltrates. Nature. 2018;557(7706):575–9. Epub 2018/05/18. 10.1038/s41586-018-0130-2 . 29769722

[17] Britanova OV, Putintseva EV, Shugay M, Merzlyak EM, Turchaninova MA, Staroverov DB, et al Age-related decrease in TCR repertoire diversity measured with deep and normalized sequence profiling. J Immunol. 2014;192(6):2689–98. Epub 2014/02/11. 10.4049/jimmunol.1302064 . 24510963

[18] Seay HR, Yusko E, Rothweiler SJ, Zhang L, Posgai AL, Campbell-Thompson M, et al Tissue distribution and clonal diversity of the T and B cell repertoire in type 1 diabetes. JCI Insight. 2016;1(20):e88242 Epub 2016/12/13. 10.1172/jci.insight.88242 27942583 PMC5135280

[19] Dandekar S, Wijesuriya H, Geiger T, Hamm D, Mathern GW, Owens GC. Shared HLA Class I and II Alleles and Clonally Restricted Public and Private Brain-Infiltrating alphabeta T Cells in a Cohort of Rasmussen Encephalitis Surgery Patients. Front Immunol. 2016;7:608 Epub 2017/01/10. 10.3389/fimmu.2016.00608 28066418 PMC5165278

[20] Schneider-Hohendorf T, Mohan H, Bien CG, Breuer J, Becker A, Gorlich D, et al CD8(+) T-cell pathogenicity in Rasmussen encephalitis elucidated by large-scale T-cell receptor sequencing. Nat Commun. 2016;7:11153 Epub 2016/04/05. 10.1038/ncomms11153 27040081 PMC4822013

[21] Ko TM, Chung WH, Wei CY, Shih HY, Chen JK, Lin CH, et al Shared and restricted T-cell receptor use is crucial for carbamazepine-induced Stevens-Johnson syndrome. J Allergy Clin Immunol. 2011;128(6):1266–76 e11. Epub 2011/09/20. 10.1016/j.jaci.2011.08.013 . 21924464

[22] Rieux-Laucat F, Bahadoran P, Brousse N, Selz F, Fischer A, Le Deist F, et al Highly restricted human T cell repertoire in peripheral blood and tissue-infiltrating lymphocytes in Omenn's syndrome. J Clin Invest. 1998;102(2):312–21. Epub 1998/07/17. 10.1172/JCI332 9664072 PMC508889

[23] Marcou Q, Mora T, Walczak AM. High-throughput immune repertoire analysis with IGoR. Nat Commun. 2018;9(1):561 Epub 2018/02/10. 10.1038/s41467-018-02832-w 29422654 PMC5805751

[24] Tickotsky N, Sagiv T, Prilusky J, Shifrut E, Friedman N. McPAS-TCR: a manually curated catalogue of pathology-associated T cell receptor sequences. Bioinformatics. 2017;33(18):2924–9. 10.1093/bioinformatics/btx286 . 28481982

[25] Springer I, Besser H, Tickotsky-Moskovitz N, Dvorkin S, Louzoun Y. Prediction of Specific TCR-Peptide Binding From Large Dictionaries of TCR-Peptide Pairs. Front Immunol. 2020;11:1803 Epub 2020/08/25. 10.3389/fimmu.2020.01803 32983088 PMC7477042

[26] Chao A, Chazdon RL, Colwell RK, Shen TJ. Abundance-based similarity indices and their estimation when there are unseen species in samples. Biometrics. 2006;62(2):361–71. 10.1111/j.1541-0420.2005.00489.x . 16918900

[27] Ritvo PG, Saadawi A, Barennes P, Quiniou V, Chaara W, El Soufi K, et al High-resolution repertoire analysis reveals a major bystander activation of Tfh and Tfr cells. Proc Natl Acad Sci U S A. 2018;115(38):9604–9. Epub 2018/08/31. 10.1073/pnas.1808594115 30158170 PMC6156623

[28] Priel A, Gordin M, Philip H, Zilberberg A, Efroni S. Network Representation of T-Cell Repertoire- A Novel Tool to Analyze Immune Response to Cancer Formation. Front Immunol. 2018;9:2913 Epub 2019/01/09. 10.3389/fimmu.2018.02913 30619277 PMC6297828

[29] Baker BM, Scott DR, Blevins SJ, Hawse WF. Structural and dynamic control of T-cell receptor specificity, cross-reactivity, and binding mechanism. Immunol Rev. 2012;250(1):10–31. Epub 2012/10/11. 10.1111/j.1600-065X.2012.01165.x 23046120

[30] Ostroumov D, Fekete-Drimusz N, Saborowski M, Kühnel F, Woller N. CD4 and CD8 T lymphocyte interplay in controlling tumor growth. Cell Mol Life Sci. 2018;75(4):689–713. Epub 2017/10/14. 10.1007/s00018-017-2686-7 29032503 PMC5769828

[31] Erickson RP. Creating animal models of genetic disease. Am J Hum Genet. 1988;43(5):582–6. 3055974 PMC1715532

[32] Gattinoni L, Speiser DE, Lichterfeld M, Bonini C. T memory stem cells in health and disease. Nat Med. 2017;23(1):18–27. 10.1038/nm.4241 28060797 PMC6354775

[33] Salio M, Silk JD, Jones EY, Cerundolo V. Biology of CD1- and MR1-restricted T cells. Annu Rev Immunol. 2014;32:323–66. Epub 2014/01/31. 10.1146/annurev-immunol-032713-120243 . 24499274

[34] Bendelac A, Savage PB, Teyton L. The biology of NKT cells. Annu Rev Immunol. 2007;25:297–336. 10.1146/annurev.immunol.25.022106.141711 . 17150027

[35] Kronenberg M, Engel I. On the road: progress in finding the unique pathway of invariant NKT cell differentiation. Curr Opin Immunol. 2007;19(2):186–93. Epub 2007/02/15. 10.1016/j.coi.2007.02.009 . 17303398

[36] MacDonald HR, Mycko MP. Development and selection of Valpha l4i NKT cells. Curr Top Microbiol Immunol. 2007;314:195–212. . 17593662

[37] Treiner E, Lantz O. CD1d- and MR1-restricted invariant T cells: of mice and men. Curr Opin Immunol. 2006;18(5):519–26. Epub 2006/07/25. 10.1016/j.coi.2006.07.001 . 16870416

[38] Kawachi I, Maldonado J, Strader C, Gilfillan S. MR1-restricted V alpha 19i mucosal-associated invariant T cells are innate T cells in the gut lamina propria that provide a rapid and diverse cytokine response. J Immunol. 2006;176(3):1618–27. 10.4049/jimmunol.176.3.1618 . 16424191

[39] Seddon B, Yates AJ. The natural history of naive T cells from birth to maturity. Immunol Rev. 2018;285(1):218–32. 10.1111/imr.12694 . 30129206

[40] Gossel G, Hogan T, Cownden D, Seddon B, Yates AJ. Memory CD4 T cell subsets are kinetically heterogeneous and replenished from naive T cells at high levels. Elife. 2017;6 Epub 2017/03/10. 10.7554/eLife.23013 28282024 PMC5426903

[41] van den Broek T, Borghans JAM, van Wijk F. The full spectrum of human naive T cells. Nat Rev Immunol. 2018;18(6):363–73. 10.1038/s41577-018-0001-y . 29520044

[42] Crotty S. Do Memory CD4 T Cells Keep Their Cell-Type Programming: Plasticity versus Fate Commitment? Complexities of Interpretation due to the Heterogeneity of Memory CD4 T Cells, Including T Follicular Helper Cells. Cold Spring Harb Perspect Biol. 2018;10(3). Epub 2018/03/01. 10.1101/cshperspect.a032102 28432129 PMC5830898

[43] Owen JA, Punt J, Stranford SA, Jones PP. Kuby immunology. Eighth edition ed. New York: W.H. Freeman, Macmillan Learning; 2018 1 volume (various pagings) p.

[44] Wooldridge L, Ekeruche-Makinde J, van den Berg HA, Skowera A, Miles JJ, Tan MP, et al A single autoimmune T cell receptor recognizes more than a million different peptides. J Biol Chem. 2012;287(2):1168–77. Epub 2011/11/22. 10.1074/jbc.M111.289488 22102287 PMC3256900

[45] Roth DB, Roth SY. Unequal access: regulating V(D)J recombination through chromatin remodeling. Cell. 2000;103(5):699–702. Epub 2000/12/15. 10.1016/s0092-8674(00)00173-2 . 11114326

[46] Bailey MH, Tokheim C, Porta-Pardo E, Sengupta S, Bertrand D, Weerasinghe A, et al Comprehensive Characterization of Cancer Driver Genes and Mutations. Cell. 2018;174(4):1034–5. Epub 2018/08/11. 10.1016/j.cell.2018.07.034 . 30096302 PMC8045146

[47] Pogorelyy MV, Fedorova AD, McLaren JE, Ladell K, Bagaev DV, Eliseev AV, et al Exploring the pre-immune landscape of antigen-specific T cells. Genome Med. 2018;10(1):68 Epub 2018/08/25. 10.1186/s13073-018-0577-7 30144804 PMC6109350

[48] Ritmahan W, Kesmir C, Vroomans RMA. Revealing factors determining immunodominant responses against dominant epitopes. Immunogenetics. 2020;72(1–2):109–18. Epub 2019/12/06. 10.1007/s00251-019-01134-9 31811313 PMC6971151

[49] Hui L, Chen Y. Tumor microenvironment: Sanctuary of the devil. Cancer Lett. 2015;368(1):7–13. Epub 2015/08/16. 10.1016/j.canlet.2015.07.039 . 26276713

[50] Shugay M, Bagaev DV, Zvyagin IV, Vroomans RM, Crawford JC, Dolton G, et al VDJdb: a curated database of T-cell receptor sequences with known antigen specificity. Nucleic Acids Res. 2018;46(D1):D419–D27. Epub 2017/10/05. 10.1093/nar/gkx760 28977646 PMC5753233

[51] Guy CT, Webster MA, Schaller M, Parsons TJ, Cardiff RD, Muller WJ. Expression of the neu protooncogene in the mammary epithelium of transgenic mice induces metastatic disease. Proc Natl Acad Sci U S A. 1992;89(22):10578–82. Epub 1992/11/15. 10.1073/pnas.89.22.10578 1359541 PMC50384

[52] Bolotin DA, Poslavsky S, Mitrophanov I, Shugay M, Mamedov IZ, Putintseva EV, et al MiXCR: software for comprehensive adaptive immunity profiling. Nat Methods. 2015;12(5):380–1. Epub 2015/04/30. 10.1038/nmeth.3364 . 25924071

[53] Vander Heiden JA, Yaari G, Uduman M, Stern JN, O'Connor KC, Hafler DA, et al pRESTO: a toolkit for processing high-throughput sequencing raw reads of lymphocyte receptor repertoires. Bioinformatics. 2014;30(13):1930–2. Epub 2014/03/13. 10.1093/bioinformatics/btu138 24618469 PMC4071206

[54] Kivioja T, Vaharautio A, Karlsson K, Bonke M, Enge M, Linnarsson S, et al Counting absolute numbers of molecules using unique molecular identifiers. Nat Methods. 2012;9(1):72–4. Epub 2011/11/22. 10.1038/nmeth.1778 . 22101854

[55] Yaari G, Kleinstein SH. Practical guidelines for B-cell receptor repertoire sequencing analysis. Genome Med. 2015;7(1):121 Epub 2015/11/22. 10.1186/s13073-015-0243-2 26589402 PMC4654805

