# Supplementary figures and images for: Breast cancer is marked by specific, Public T-cell receptor CDR3 regions shared by mice and humans
Source: PLoS Comput Biol. 2021 Jan 19;17(1):e1008486. doi: 10.1371/journal.pcbi.1008486 (PMC7846026; doi:10.1371/journal.pcbi.1008486)

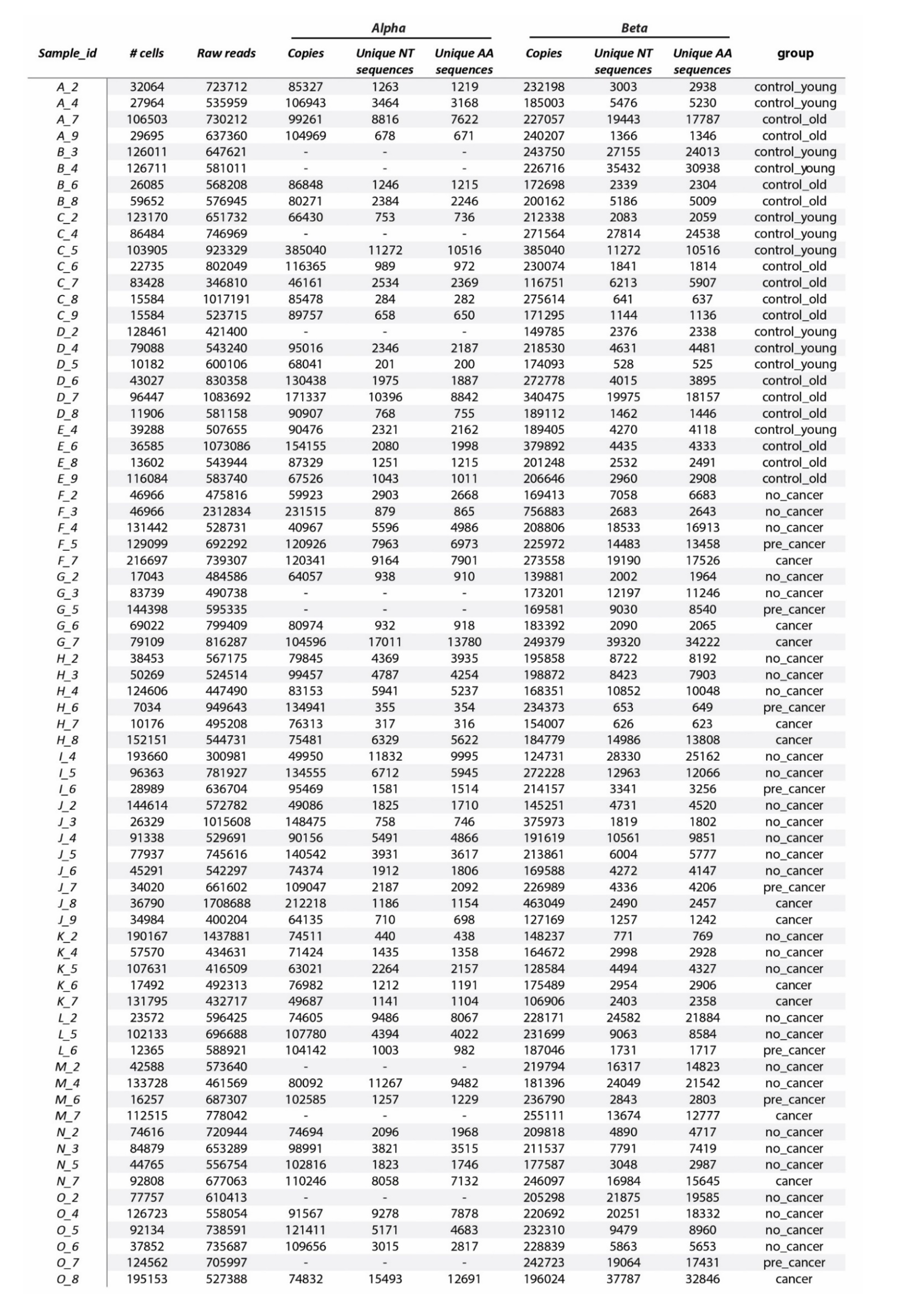

Supplement: S3 Table — Rows are the different samples and columns are the different stages. (TIF) [file pcbi.1008486.s003.tif]

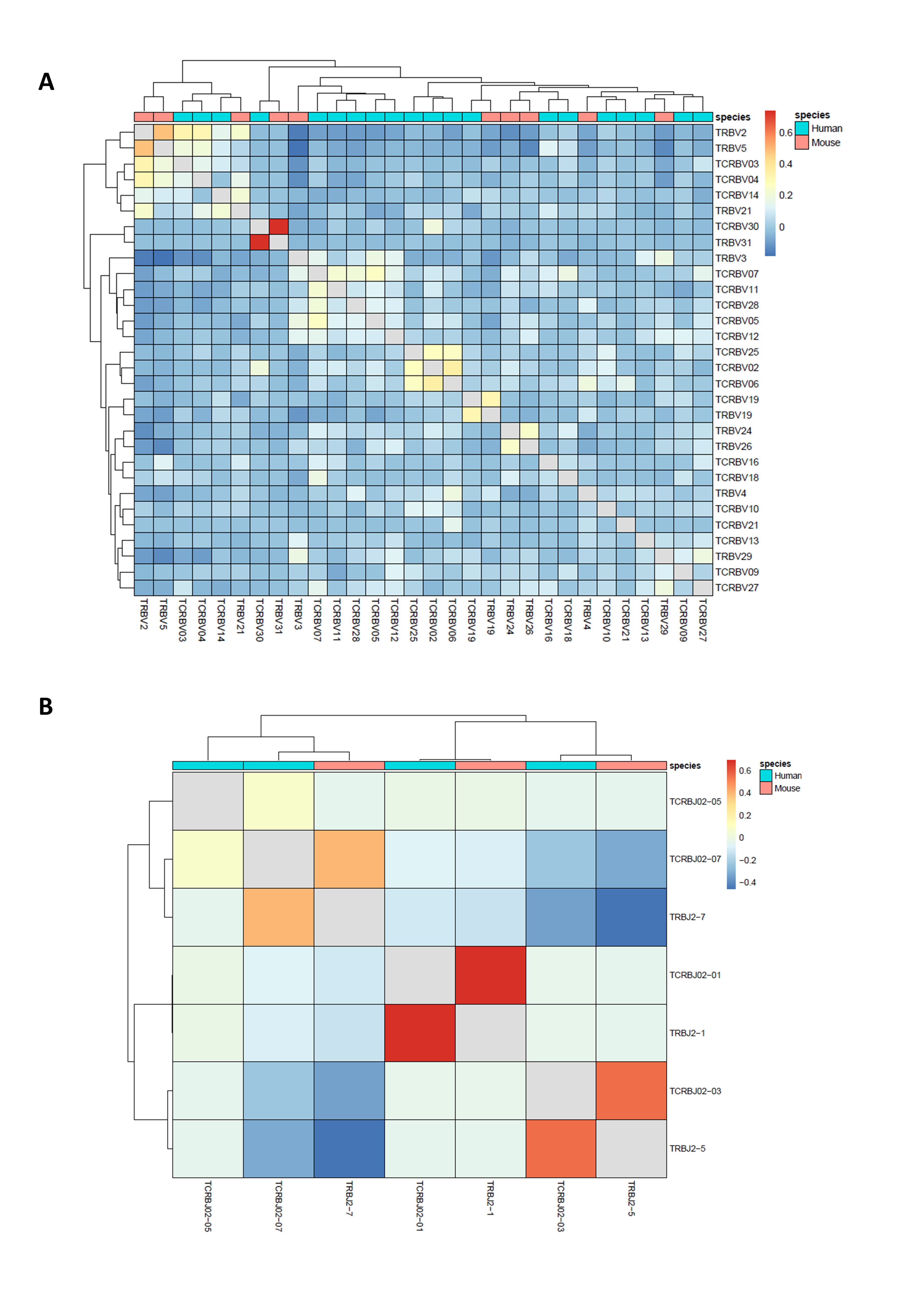

Supplement: S6 Fig — The correlations between the different Vs (A) and Js (B) usage of the tumor-associated cross-species clones. (TIF) [file pcbi.1008486.s010.TIF]

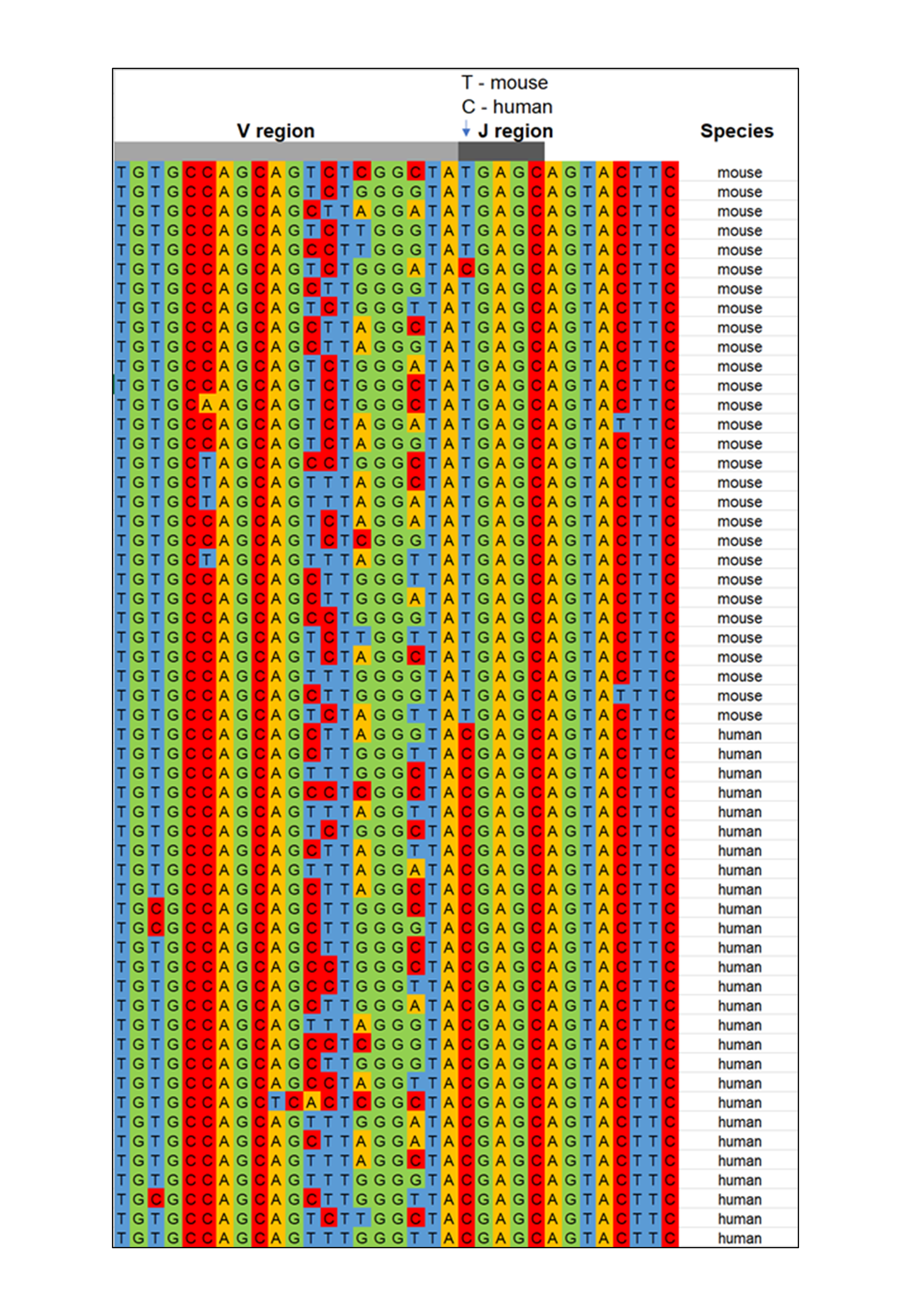

Supplement: S7 Fig — The NT display of the AA compositions demonstrated in Fig 4C. The figure reveals the recombination effect: the same AA sequences were built on a combination of 9 different TRBVs (-2,3,4,5,19,21,24,26,29), while TRBJ2-7, both for mouse and for human, was used. The colored bars represent the NT sequences encoding to these AA sequences. The colored bars represent the NT sequences encoded to these AA sequences. Each color represents different nucleotide: T–blue; G–yellow; C–green; A–red. (TIF) [file pcbi.1008486.s011.TIF]
